# Carbonylative Coupling of 1‑Iodoglucal and Amino Acids: Access to New Fluorescent Sugar Amino Acids

**DOI:** 10.1021/acsomega.5c04865

**Published:** 2025-07-15

**Authors:** Pamela M. Silva, Mônica F. Z. J. Toledo, Flávia Manarin, Daniel C. Pimenta, Nicaely M. O. Pereira, Erick. L. Bastos, Vinicius M. da Silva, Milene M. Hornink, Hélio A. Stefani

**Affiliations:** † Centro Universitário São Camilo, São Paulo, São Paulo 05508-900, Brazil; ‡ Departamento de Farmácia, Faculdade de Ciências Farmacêuticas, Universidade de São Paulo, São Paulo, São Paulo 05508-900, Brazil; § Centrode Engenharias e Ciências Exatas, Unioeste Toledo, Paraná 85903-000, Brazil; ∥ Instituto Butantan, São Paulo, São Paulo 05508-900, Brazil; ⊥ Departamento de Química Fundamental, Instituto de Química, Universidade de São Paulo, São Paulo, São Paulo 05508-900, Brazil

## Abstract

This study reports on the development of a palladium-catalyzed carbonylative coupling reaction for synthesizing glucal amino acids and fluorescent amino acid derivatives. A metal carbonyl was employed as a CO surrogate, avoiding the use of CO gas. Utilizing Pd­(OAc)_2_ and triphenylphosphine, methyl esters of L-amino acids were coupled with 1-iodoglucal under optimized reaction conditions. Yields of up to 65% were achieved. The methodology was applied to amino acids with different side chains. Notably, the synthesized fluorescent derivatives containing stilbene moieties exhibited distinct absorption and emission properties with high fluorescence quantum yields.

## Graphical abstract

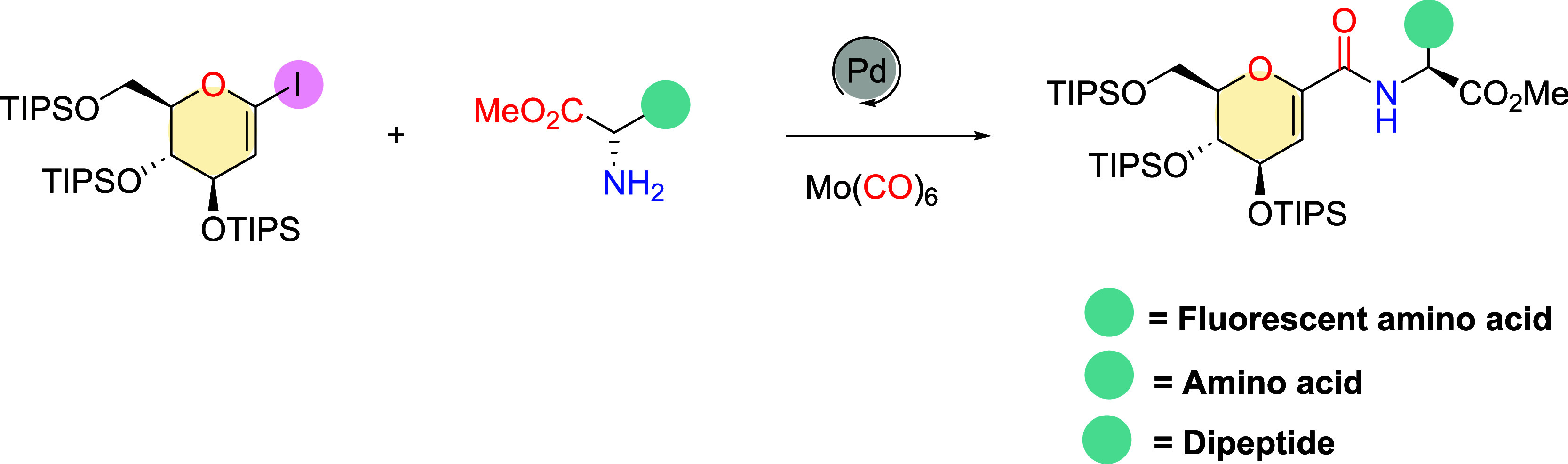

## Introduction

Lasky’s discovery in 1992 of the role of cell surface glycoproteins in the inflammatory process opened a new gateway for the development of synthetic approaches aimed at the construction of sugar-linked amino acids.^1^ This is especially true for preparing non-natural sugar amino acids, in which the α-amino acid group is tethered to the anomeric carbon atom of the sugar moiety.^2^ These carbon-linked *C*-glycosyl amino acids exhibit increased conformational rigidity; free amino acids or peptides typically have many conformational degrees of freedom.^3^ Furthermore, the *C*-tethered analogues are enzymatically and metabolically resistant.^2^ Collectively, these features position the fused sugar amino acids as a promising scaffold for the design of bioactive molecules.^4^

Although sugar amino acids occur naturally, only a few known natural *C*-glycosyl amino acids exist. Important examples are α-*C*-mannosyl tryptophan (**1**, Scheme 1),^5^ whose synthesis has been pursued by several groups,^6^ and hydantocidin (**2**), a phytotoxin isolated from *Streptomyces hygroscopicus*,^7^ whose derivatives show glycosidase inhibitor activity.^8^

**Scheme 1:**
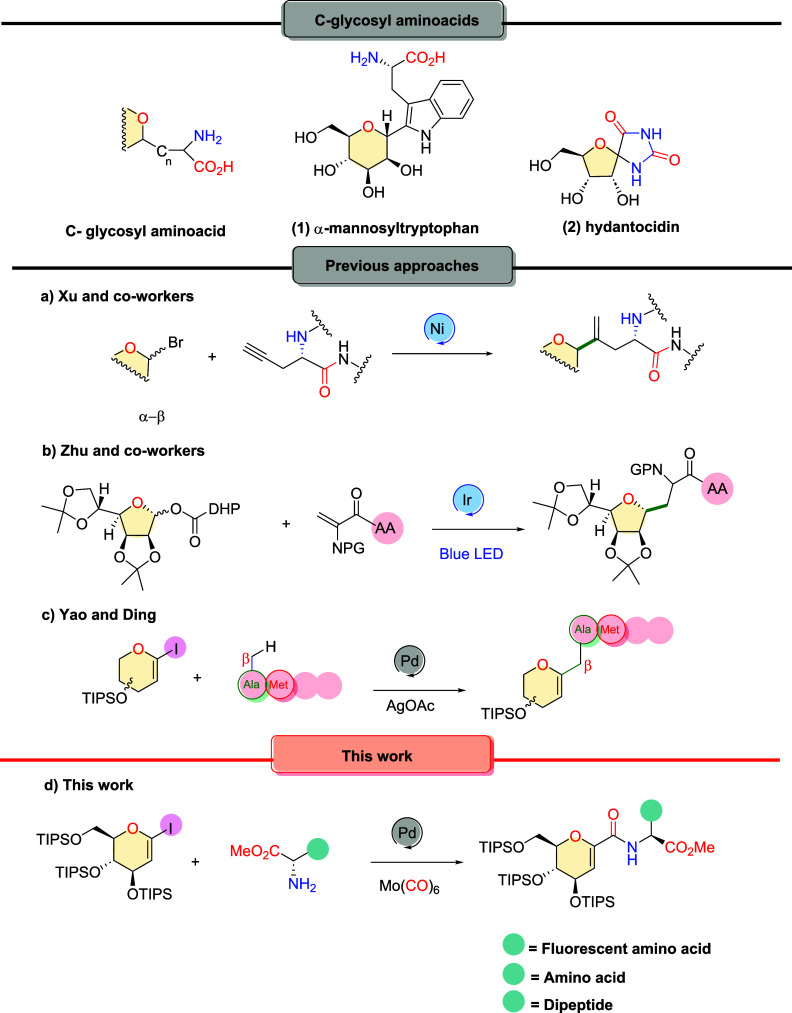
Approaches for Linking Sugars and Amino Acids: (a–c) Previous Approaches; (d) This Work

Considering the ultimate application of glycosyl amino acids as foundational building blocks for the synthesis of glycopeptide mimic libraries,^2^ different methodologies have been proposed for linking amino acids and sugars.^9^ Xu and coworkers demonstrated that amino acids or peptides containing an alkynyl pendant and glycosyl bromides can be selectively coupled in a regio- and stereoselective manner under Ni catalysis, yielding metabolically stable vinyl *C*-glycosyl amino acids and peptides (Scheme 1a).^10^ In 2023, Zhu and coworkers published the first visible-light photoredox-catalyzed C­(sp^3^)-glycosylation of redox-active glycosyl dihydropyridine (DHP) esters with dehydroalanine (Dha) derivatives via anomeric C–O bond homolysis, enabling the concise synthesis of alkyl *C*-glycoamino acids and *C*-glycosyl peptides (Scheme 1b).^11^ Recently, Yao and Ding demonstrated an inverse approach to peptide glycosylation, reporting a Pd­(II)-catalyzed methionine-directed β*-*C­(sp^3^)–H glycosylation of peptides with 1-iodoglycals to construct *C*-glycosyl glycopeptides (Scheme 1c).^12^

In the field of chemical biology, the emergence of new labeling strategies for fluorescence-based techniques has created a need for novel methodologies to construct non-natural fluorescent amino acids.^13^ The carbonylative coupling reaction is frequently employed to couple aryl halides and amino acids.^14a^ Our group has studied carbonylative coupling reactions with sugars and recently reported the synthesis of *C*2-branched glycosides bearing an amino acid group.^13^ Compared with the preparation of the native *O*/*N*- or artificial *S*/*Se*-glycosyl peptides, the synthesis of *C*-glycosyl peptides is more challenging due to the lower nucleophilicity of the carbon center, highlighting a gap in this field that remains underexplored. Peptide-based therapeutics represent a key area in the development of new pharmaceuticals.^15^ Consequently, developing synthetic routes to couple amino acids with diverse moieties without relying on acid chlorides, anhydrides, or stoichiometric coupling reagents such as *N*,*N*′*-*diisopropylcarbodiimide (DIC), 1-ethyl-3-(3-(dimethylamino)­propyl)­carbodiimide (EDCI), or hexafluorophosphate de (dimethylamino)-*N*,*N*-dimethyl­(3H-[1,2,3]­triazolo­[4,5-*b*]­pyridin-3-yloxy)­methaniminium (HATU) is desirable.^16^

Herein, we investigated the palladium-catalyzed carbonylative coupling of l-amino acid esters and 1-iodoglucal by employing a metal carbonyl as a CO surrogate (Scheme 1d), thereby eliminating the need to handle toxic CO gas. This approach enabled the synthesis of novel fluorescent amino acid analogs, whose photochemical properties were subsequently examined.

## Results and Discussion

Triisopropylsilyl (TIPS)-protected 1-iodoglucal (**1a**) and l-amino acid methyl esters (**2a**) were used as starting materials for synthesizing glucal amino acids (Figure 1). l-Amino acids were converted into the corresponding methyl esters by reactions with thionyl chloride (SOCl_2_) in anhydrous methanol.^17^ 1-Iodoglucal was prepared in two steps. The free hydroxyl groups of d-glucal were protected via silylation with triisopropylsilyl chloride (TIPSCl) and imidazole in DMF.^18^ Subsequently, the silylated glucal vinyl anion was generated by adding *t*-BuLi in THF at −78 °C, followed by a reaction with diiodoethane (C_2_H_4_I_2_), yielding the desired 1-iodoglucal.^19^

**Figure 1:**
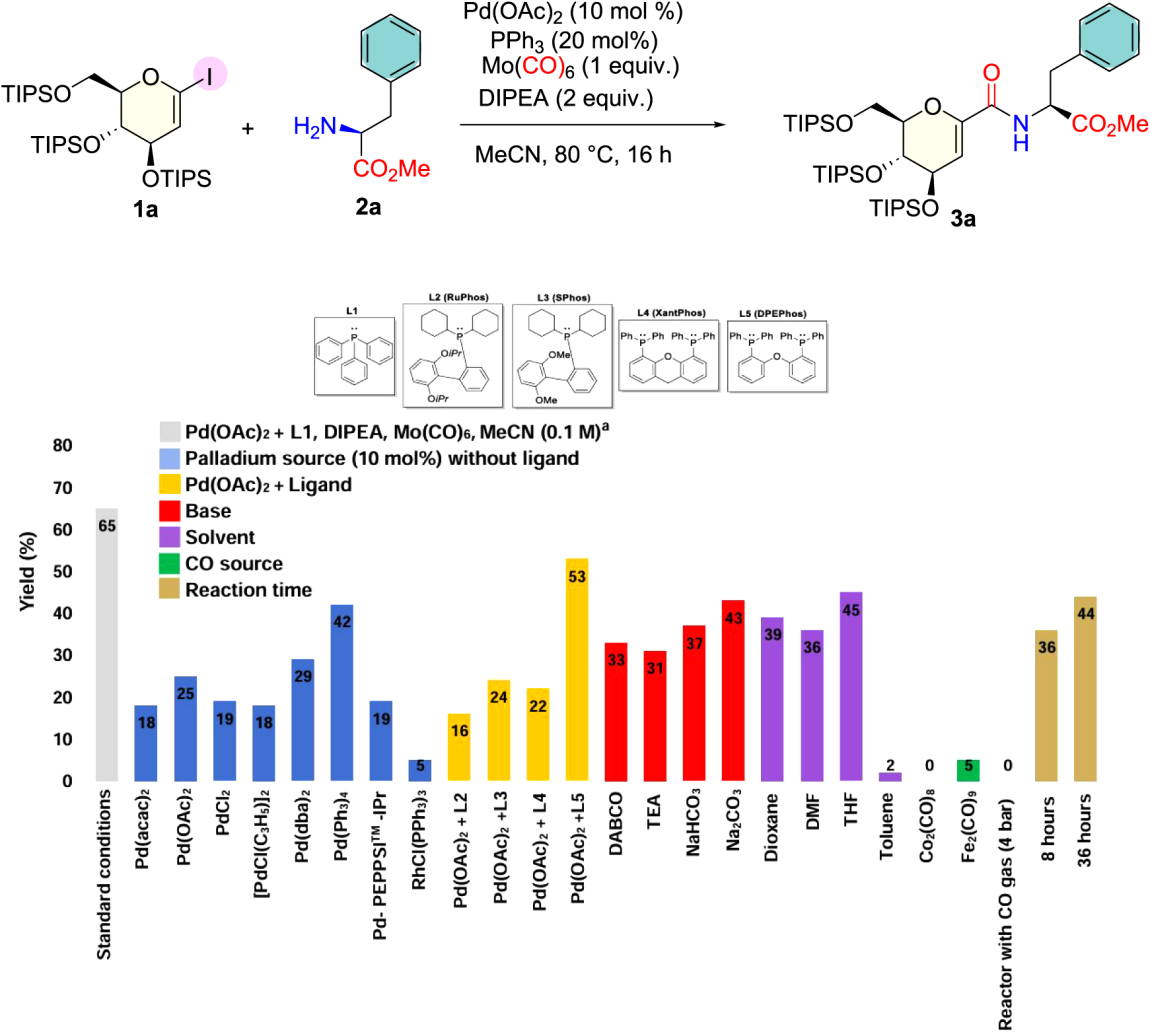
Synthesis of glucal amino acids. ^a^ Reactions conditions: **1a** (0.1 mmol; 1.0 equiv); **2a** (0.12; 1.2 equiv); Pd­(OAc)_2_ (10 mol %); PPh_3_ (20 mol %); DIPEA (0.2 mmol; 2.0 equiv); Mo­(CO)_6_ (1.0 equiv) and their corresponding yields for the coupling of 1-iodoglucal and l-phenylaniline methyl ester.

With the starting materials in hand, we systematically explored a range of reaction conditions for the coupling between 1-iodoglucal (**1a**, 1 equiv) and l-phenylaniline methyl ester as the nucleophile (**2a**, 1.2 equiv). For the standard reaction condition, we employed Mo­(CO)_6_ (2 equiv) as a solid CO source,^20a^ diisopropylethylamine (DIPEA, 2 equiv) as the base, Pd­(OAc)_2_ (10 mol %) and triphenylphosphine (20 mol %, L1, Figure 1) as the catalytic system, and acetonitrile as the solvent. The reaction time was 16 h, and temperature was 80 °C. Under these conditions, compound **3a** was obtained with a 65% yield, as determined by ^1^H NMR using trichloroethylene as an internal standard (IS). To be sure about the role of the phosphine ligands in the catalytic system, we tested some other palladium sources without the addition of any ligands, including Pd(0) catalysts, such as Pd­(dba)_2_. As depicted in Figure 1, the yields were decreased, showcasing the rule of the phosphine ligand in the reaction. We also applied two Pd(0) catalysts, Pd­(dba)_2_ and Pd­(PPh_3_)_4_. Both provided lower yields of the desired product, 29% and 42%, respectively (Figure 1). Using another metal (i.e., a rhodium complex) resulted in a trace amount of product. Next, we added some ligands, but only Xantphos exhibited a good yield (53%) (Figure 1). However, the yield was still lower than with the standard conditions. The use of different bases (such as DABCO and Na_2_CO_3_) also led to lower yields (Figure 1; 33% and 43%, respectively), revealing that DIPEA was necessary for achieving higher yields. Different solvents were also explored, and all, including THF (Figure 1, 45%), resulted in lower yields. We also explored other sources of CO gas, including different metal carbonyls, such as Co_2_(CO)_8_ (Figure 1). In this example, only the starting material was observed. Applying a longer reaction time (36 h) (Figure 1) also led to a decrease in the reaction yield. Finally, attempting to perform the reaction in a shortened time of 8 h did not improve the reaction yield.

Next, using the standard reaction conditions, we explored the carbonylative coupling reaction with different amino acid methyl esters. The isolated yield of compound **3a** was 64% (Scheme 2). We then applied l-tyrosine methyl ester, and although the former is structurally related to l-phenylalanine, the presence of the hydroxyl group led to a decrease in the isolated yield, as depicted in Scheme 2, **3b**. We noticed that a double coupling was occurring, and the phenolic hydroxyl group was acting as a nucleophile. This observation led us to protect the hydroxyl group with *tert*-butyl­(dimethyl)­silyl (TBS). However, using the (*tert*-butyldimethylsilyl ether) OTBS-protected amino acid led to a lower isolated yield (17%) of compound **3c** (Scheme 2). Therefore, we decided to explore another aromatic amino acid. The use of l-tryptophan methyl ester provided a 63% yield of the desired isolated product (Scheme 2, **3d)**. Next, we decided to evaluate the use of l-amino acids with nonpolar aliphatic side chains. Our first attempt was with l-valine methyl ester, and our standard conditions were used. This experiment led to a considerably low yield of 27% of the desired coupling product (Scheme 2, **3e)**. Although we thoroughly exploited conditions to perform a general coupling reaction with high yields, our standard reaction conditions were effectively applied to a few l-amino acid methyl ester examples. We believe that the low nucleophilicity of the α-amino group, the presence of chemically different side chains, and steric hindrance led to a lack of reproducibility in the reactivity.^14b^

**Scheme 2:**
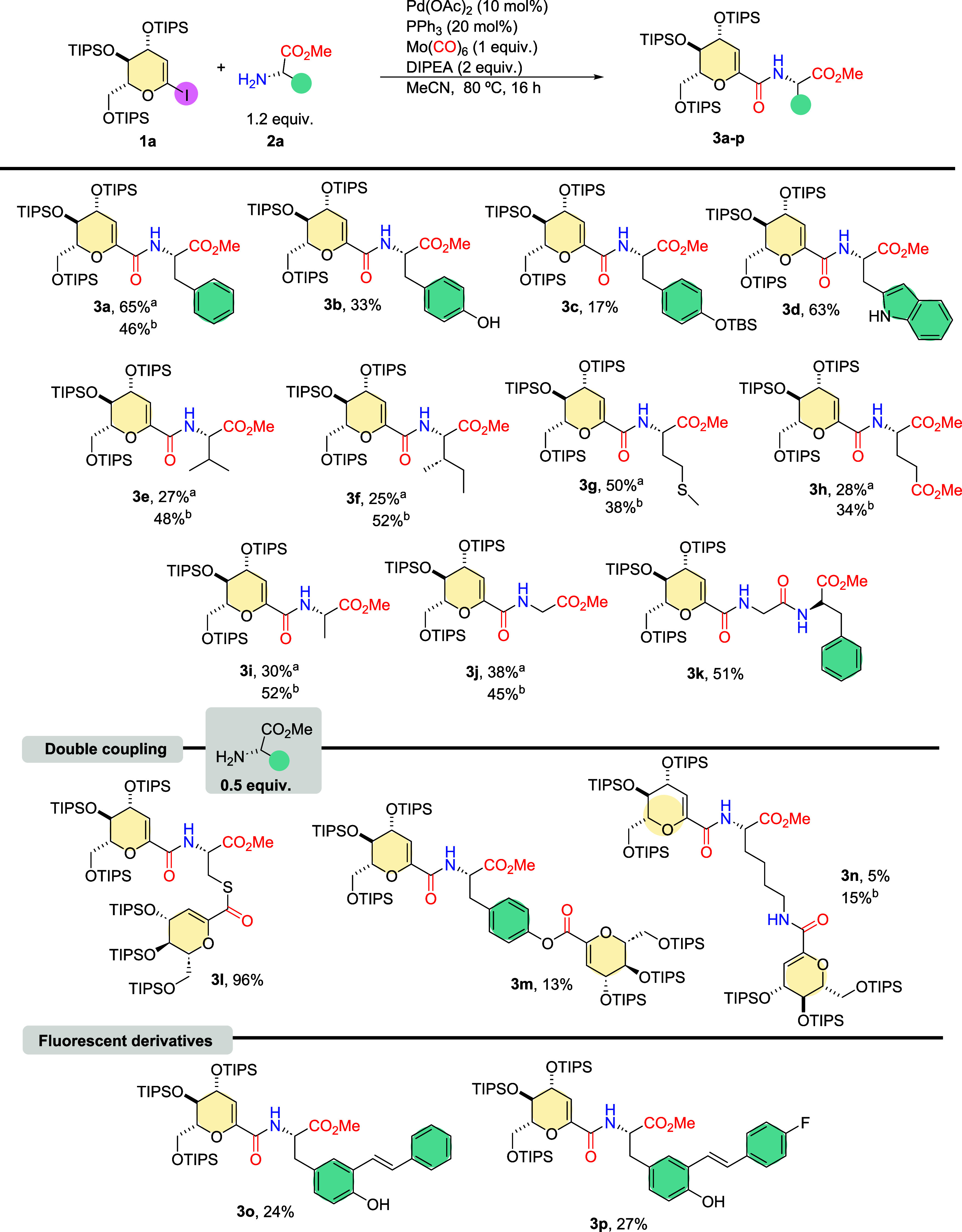
Amino Acid Scope^a^

a Reaction conditions: **1a** (0.1 mmol, 1.0 equiv), **2a** (0.12 mmol, 1.2 equiv), Pd­(OAc)_2_ (10 mol %), PPh_3_ (20 mol %), DIPEA (0.2 mmol, 2.0 equiv), Mo­(CO)_6_ (0.1 mmol, 1.0 equiv), MeCN (0.1 M) ^b^Pd­(PPh_3_)_4_ (10 mol %) without phosphine.

We decided to use the other catalytic system that performed well with our model substrate l-phenylalanine and then applied palladium-tetrakis (Pd­(PPh_3_)_4_) (10 mol %). To our surprise, this condition led to an isolated yield of 48% (**3e**). Similar lower isolated yields were observed with l-isoleucine, l-glutamic acid, l-valine, and l-glycine methyl esters. We also performed coupling reactions with palladium-tetrakis, which led to an increase in the isolated yields (Scheme 2, **3f, 3h, 3i, 3j)**. The use of l-methionine methyl ester under the standard conditions led to a good, isolated yield (Scheme 2, **3g**). When the reaction was performed with palladium tetrakis, the yield decreased.

Then, we performed some double coupling reactions, intentionally changing the stoichiometry of the reaction and using an excess of 1-iodoglucal (2 equiv). First, we evaluated the l-cysteine methyl ester. Under these conditions, the isolated yield of product **3l** was 96% (Scheme 2). Second, we tested l-lysine methyl ester, but the yield was only 5%. Considering that the aforementioned condition with palladium-tetrakis improved the performance for most amino acids containing an aliphatic side chain, we applied this condition to this example, resulting in an increased yield of 15% for **3n** (Scheme 2). Finally, we applied the same stoichiometry conditions to increase the ratio of double coupling with l-tyrosine methyl ester. However, we isolated only 13% of product **3l** (Scheme 2).

Furthermore, to broaden the potential applications of this approach, we extended the example to a dipeptide. Specifically, we performed the carbonylative coupling reaction on the glycine-phenylalanine dipeptide, achieving a satisfactory yield of 51% for **3k** (Scheme 2).

Then, we turned our attention to carbonylative coupling using fluorescent amino acid derivatives. We applied a compound that can be prepared from 3-iodo-l-tyrosine, performing the coupling with trifluoroalkylborates or boronic acids.^21a^ The fluorescent derivatives with a stilbene moiety were prepared with an isolated yield of 24% (compound **3o,** Scheme 2). The derivative with a fluor substituted in position 4 of the stilbene ring (compound **3p,** Scheme 2) had an isolated yield of 27%.

Next, synthetic transformations were carried out to demonstrate the potential applications of the obtained products. A scale-up reaction was performed using tryptophan as the amino acid precursor. The carbonylative coupling reaction at a 1 mmol scale afforded the coupling product with a 50% yield (Scheme 3), showing only a slight reduction compared to the smaller-scale reaction. Subsequently, other 1-iodoglycal was tested in the aminocarbonylation conditions, the 1-iodo-L-rhamnal, synthesized according to the procedures outlined in the literature.^22^ As shown in Scheme 3, compound **4** was isolated with a 40% yield. And finally, deprotection of the intermediate was achieved using tetrabutylammonium fluoride (TBAF) in THF as the solvent. The resulting hydroxylated *C*-glycoside amino acid had a 77% yield (Scheme 3).

**Scheme 3:**
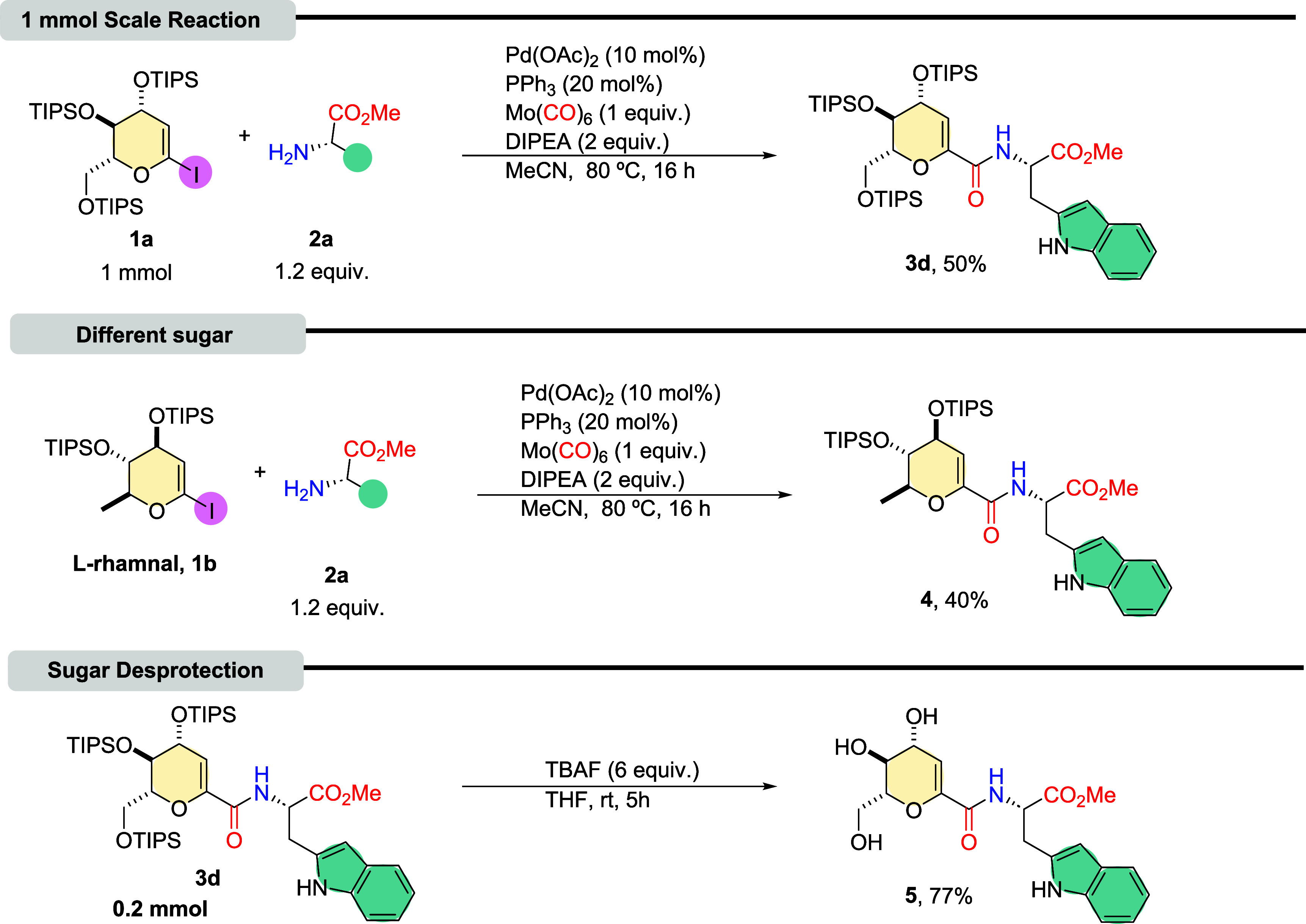
Synthetic Applications[Fn sch3-fn2]

a Reaction conditions for sugar deprotection: **3d** (0.2 mmol, 1.0 equiv), TBAF (1.2 mmol, 6 equiv), THF (6 mL).

The absorption and fluorescence spectra of **3o** were acquired in CHCl_3_ (Figure 2a) because the fluorescence profiles of *o*-hydroxystilbene-substituted tyrosine derivatives have been shown to be insensitive to solvent effects.^23^ The absorption profile (λ_max_ at ∼290 and 325 nm), singlet energies (*E*_S_), and fluorescence quantum yields (Φ_f_) of compound **3o** (i.e., 315 kJ mol^–1^ and 0.14 ± 0.02 (14%), respectively), are in agreement with the data reported previously for *o-*hydroxystilbene-substituted tyrosine peptides.^23^ The emission profile (λ_*f*_ at ∼380 nm) is independent of the excitation wavelength and could be assigned to the lowest ^1^π, π* state of the stilbene core. As previously reported by our group, the fluorescence of **3o** results from the stilbene core, and the tyrosine-triazole moiety has a minor effect on the fluorescence of the *o*-hydroxystilbene-substituted tyrosine derivatives.^23^ The high fluorescence quantum yield of **3o** in CHCl_3_ at room temperature compared to *p*-hydroxystilbene (5%), but not to *m-*hydroxystilbene (92%), may be related to a combination of electronic effects from the *o-*hydroxy group and steric effects that increase the C=C torsion barrier of the stilbene core.^23^

**Figure 2:**
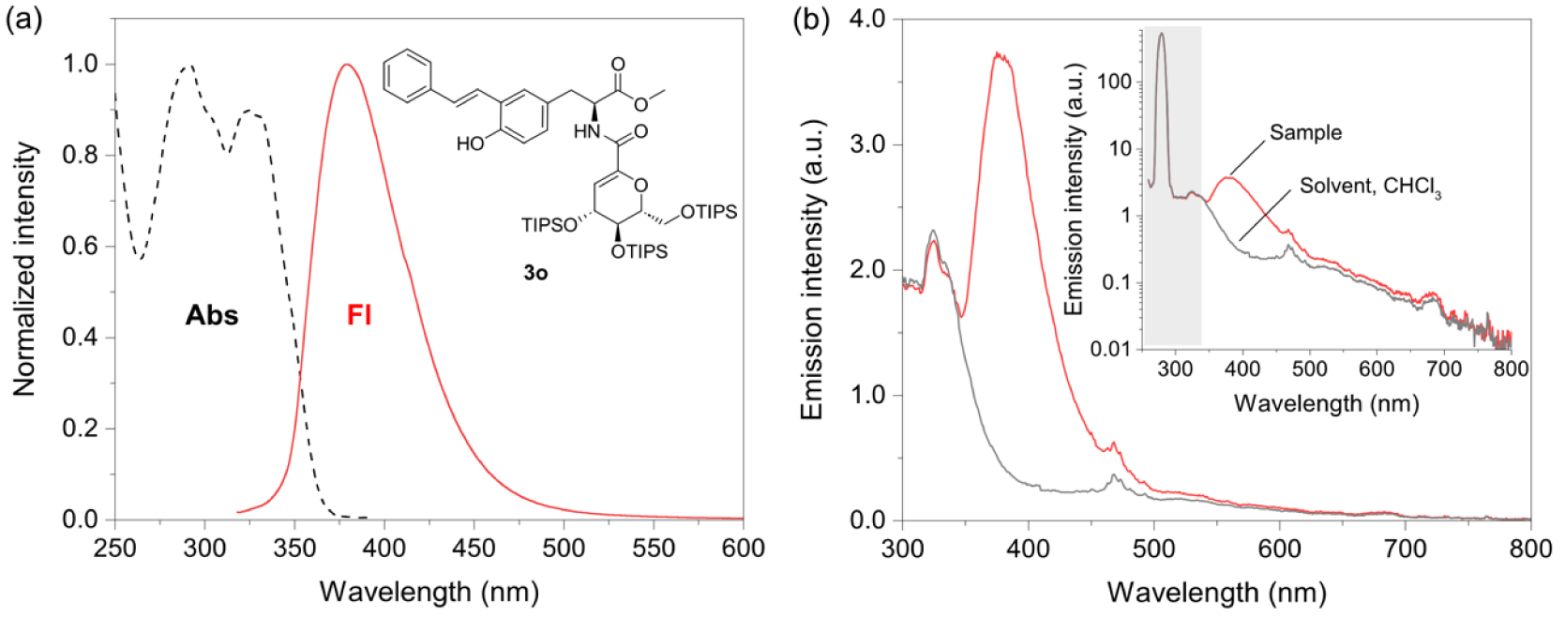
Absorption and fluorescence properties of **3o**. (a) Normalized absorption and corrected fluorescence spectra of solutions of compound **3o** in aerated CHCl_3_ at 25 °C. Excitation wavelength: 280 nm; excitation bandwidth: 10 nm; emission bandwidth: 1.47 nm. (b) The spectra of a reference scatterer (CHCl_3_, black line) and the sample (red line). The inset shows the reflectance (gray area) and emission of the sample used to calculate the Φ_f_.

## Conclusion

In conclusion, this study highlights the successful development of a palladium-catalyzed carbonylative coupling method for synthesizing glucal amino acids and fluorescent amino acid derivatives, using a metal carbonyl as a CO surrogate to avoid handling toxic CO gas. Although we strived to develop a general reaction, the presence of chemically different side chains and steric hindrance led us to perform some focused optimization, especially for the amino acids with alkyl nonpolar side chains. Additionally, the fluorescent derivatives synthesized display promising photochemical properties, including high fluorescence quantum yields, highlighting their potential for various applications in chemical biology.

## Experimental Section

### General Information

All reagents were purchased from Sigma-Aldrich, Alfa Aesar, Acros Organics, Oakwood or Fluorochem. When they were not a HPLC-grade solvents, they were purified by distillation. Other solvents, like DIPEA was also dried over CaH_2_. The *O*-TIPS protection of d-glucal/L-rhamnal and its subsequent C1 iodination was performed using protocols reported in literature.^19,22,24^ Esterification of amino acids were performed using thionyl chloride (SOCl_2_) and anhydrous methanol.^17^ The synthesis of fluorescent amino acids derivatives were previously reported by Vasconcelos et al. and Boc-deprotection reported by Suzuki et al.^21a^ The Thin Layer Chromatography was carried out using g Merck TLC 60 F254 silica gel plates and visualized under UV light (254 nm) and stained with acidic vanillin solution. Flash column chromatography was performed using silica gel with a pore size of 60 Å, 230–400 Mesh (Sigma-Aldrich, cat.# 22,719-6). Nuclear magnetic resonance (NMR) spectra were recorded in CDCl_3_ or DMSO-*d*_6_ using a Bruker DPX 300 instrument (^1^H at 300 MHz, ^13^C at 75 MHz). Chemical shifts, δ, are reported in parts per million (ppm) and are referenced to the TMS signal. ^1^H peaks are quoted to the nearest 0.01 Hz and ^13^C peaks are quoted to the nearest 0.1 Hz. The abbreviation utilized to report the peaks are s (singlet), d (doublet), t (triplet), dd (doublet of doublets), m (multiplet). High-resolution mass spectra (HRMS) were recorded on a Shimadzu ESI-TOF mass spectrometer or Shimadzu MALDI-TOF Axima Performance spectrometer. FTIR data were obtained using an Agilent Technologies Cary 630. Optical rotations were measured at 20 °C by using an Anton Paar MCP200 Polarimeter.

### General Procedure for the Synthesis of Glycals Amides (**3a**–**3p** and **4**)

To a flame-dried 5 mL reaction tube capped with a rubber septum were added 1-iodo-glycal (0.1 mmol), amino ester (0.12 mmol, 1.2 equiv), DIPEA (35 μL; 0.2 mmol, 2 equiv), palladium (^a^Pd­(OAc)_2_ (2.25 mg, 10 mol %)—PPh_3_ (5.25 mg, 20 mol %) or ^b^Pd­(PPh_3_)_4_ (11.55 mg, 10 mol %)) and Mo­(CO)_6_ (26.4 mg, 0.1 mmol, 1.0 equiv). Dry and degassed MeCN was added to the system (0.8 mL). The mixture was then stirred at 80 °C for 16 h. The resulting mixture was filtered through a sintered funnel with Celite and washed with EtOAc to remove insoluble materials. The filtrate was concentrated under reduced pressure and purified by flash column chromatography (eluent: 0 to 40% EtOAc in hexanes).

#### Methyl ((2*R*,3*R*,4*R*)-3,4-Bis­((triisopropylsilyl)­oxy)-2-(((triisopropylsilyl)­oxy)­methyl)-3,4-dihydro-2*H*-pyran-6-carbonyl)-l-phenylalaninate (**3a**)

The product was obtained as a pale-yellow oil (65%^a^–45%^b^). [α]_D_^20^ = +15 (*c* = 0.1 in CHCl_3_). ^1^H NMR (300 MHz, CDCl_3_) δ 7.18–7.15 (m, 3H), 7.07–7.02 (m, 3H), 5.98 (dd, *J* = 5.3 Hz, 1.4 Hz, 1H), 4.87–4.81 (m, 1H), 4.32–4.29 (m, 1H), 4.04–4.00 (m, 2H), 3.92 (dd, *J* = 11.4 Hz, 8.1 Hz, 1H), 3.69 (dd, *J* = 11.4 Hz, 3.8 Hz, 1H), 3.60 (s, 3H), 3.10 (dd, *J* = 13.7 Hz, 5.8 Hz, 1H), 3.02 (dd, *J* = 13.8 Hz, 5.9 Hz, 1H), 0.98–0.93 (m, 63H). ^13^C NMR (75 MHz, CDCl_3_) δ 171.4, 161.9, 143.1, 136.0, 129.4 (2C), 128.2 (2C), 127.1, 104.6, 82.1, 69.9, 65.8, 61.3, 53.4, 52.2, 38.3, 18.2–17.8 (18C), 12.6 (3C), 12.4 (3C), 12.1 (3C). IR (ν, cm^–1^) = 3296, 2846, 2771, 1687, 1631, 1601, 1452, 1413, 1315, 1300, 1022, 855, 730. HRMS (ESI- TOF) *m*/*z*: [M + Na]^+^ calc. for C_44_H_81_NO_7_Si_3_ 842.5218; found 842.5219.

#### Methyl ((2*R*,3*R*,4*R*)-3,4-Bis­((triisopropylsilyl)­oxy)-2-(((triisopropylsilyl)­oxy)­methyl)-3,4-dihydro-2*H*-pyran-6-carbonyl)-l-tyrosinate (**3b**)

The product was obtained as a pale-yellow oil (33%^a^). [α]_D_^20^ = +16 (*c* = 0.1 in CHCl_3_). ^1^H NMR (300 MHz, CDCl_3_) δ 7.17 (d, *J* = 8.0 Hz, 1H), 6.95 (d, *J* = 8.0 Hz, 2H), 6.69 (d, *J* = 8.1 Hz, 1H), 6.05 (d, *J* = 5.3 Hz, 1H), 4.90–4.83 (m, 1H), 4.40–4.37 (m, 1H), 4.13–4.07 (m, 2H), 4.00 (dd, *J* = 11.5, 8.2 Hz, 1H), 3.76 (dd, *J* = 11.5, 3.8 Hz, 1H), 3.66 (s, 3H), 3.08 (dd, *J* = 13.9, 6.1 Hz, 1H), 3.01 (dd, *J* = 13.9, 6.1 Hz, 1H), 1.06–1.01 (m, 63H). ^13^C NMR (75 MHz, CDCl_3_) δ 171.5, 162.0, 155.1, 143.0, 130.4 (2C), 127.7, 115.6 (2C), 104.8, 82.2, 69.9, 65.7, 61.3, 53.7, 52.2, 37.6, 18.2–18.0 (18C), 12.6 (3C), 12.4 (3C), 12.1 (3C). IR (ν, cm^–1^) = 3296, 2846, 2771, 1693, 1596, 1466, 1413, 1052, 1026, 855, 730. HRMS (ESI- TOF) *m*/*z*: [M + Na]^+^ calc. for C_44_H_81_NO_8_Si_3_ 858.5167; found 858.5169.

#### Methyl (*S*)-2-((2*R*,3*R*,4*R*)-3,4-Bis­((triisopropylsilyl)­oxy)-2-(((triisopropylsilyl)­oxy)­methyl)-3,4-dihydro-2*H*-pyran-6-carboxamido)-3-(4-((*tert*-butyldimethylsilyl)­oxy)­phenyl)­propanoate (**3c**)

The product was obtained as a pale-yellow oil (17%^a^). [α]_D_^20^ = +10 (c = 0.1 in CHCl_3_). ^1^H NMR (300 MHz, CDCl_3_) δ 7.12 (d, *J* = 7.4 Hz, 1H), 6.95 (d, *J* = 8.3 Hz, 2H), 6.70 (d, *J* = 8.3 Hz, 1H), 6.04 (dd, *J* = 5.3 Hz, 1.7 Hz, 1H), 4.90–4.83 (m, 1H), 4.39–4.35 (m, 1H), 4.11–4.06 (m, 2H), 3.099 (dd, *J* = 11.3 Hz, 8.0 Hz, 1H), 3.75 (dd, *J* = 11.4 Hz, 3.8 Hz, 1H), 3.65 (s, 3H), 3.07 (dd, *J* = 13.9 Hz, 5.8 Hz, 1H), 3.02 (dd, *J* = 13.9 Hz, 5.8 Hz, 1H), 1.06–1.00 (m, 63H), 0.96 (s, 9H), 0.16 (s, 6H). ^13^C NMR (75 MHz, CDCl_3_) δ 171.4, 161.9, 154.8, 143.2, 130.3 (2C), 128.7, 120.2 (2C), 104.6, 82.1, 69.9, 65.8, 61.3, 53.6, 52.2, 37.6, 25.8 (3C), 18.3–18.0 (18C), 12.6 (3C), 12.4 (3C), 12.0 (3C), −4.3 (2C). ^13^C NMR (75 MHz, CDCl_3_) δ 171.4, 161.9, 154.7, 143.1, 130.3 (2), 128.6, 120.2 (2), 104.6, 82.1, 69.9, 65.7, 61.3, 53.6, 52.2, 37.5, 25.8 (3C), 18.3–18.0 (18C), 12.5 (3C), 12.4 (3C), 12.0 (3C), −4.3 (2C). IR (ν, cm^–1^) = 3310, 2833, 2771, 1670, 1601, 1460, 1413, 1225, 1033, 855, 728. HRMS (ESI-TOF) *m*/*z*: [M + Na]^+^ calc. for C_50_H_95_NO_8_Si_4_ 972.6032; found 972.6034.

#### Methyl ((2*R*,3*R*,4*R*)-3,4-Bis­((triisopropylsilyl)­oxy)-2-(((triisopropylsilyl)­oxy)­methyl)-3,4-dihydro-2*H*-pyran-6-carbonyl)-l-tryptophanate (**3d**)

The product was obtained as a pale-yellow oil (63%^a^). [α]_D_^20^ = +11 (c = 0.1 in CHCl_3_). ^1^H NMR (300 MHz, CDCl_3_) δ 8.01 (br, 1H), 7.57 (d, *J* = 7.7 Hz, 1H), 7.25 (d, *J* = 7.6 Hz, 1H), 7.15 (td, *J* = 8.0 Hz, 1.2 Hz, 1H), 7.09 (td, *J* = 8.0 Hz, 1.1 Hz, 1H), 6.97 (d, *J* = 2.1 Hz, 1H), 6.08 (dd, *J* = 5.3 Hz, 1.5 Hz, 1H), 4.98–4.92 (m, 1H), 4.37–4.35 (m, 1H), 4.13–4.07 (m, 2H), 3.98 (dd, *J* = 11.3 Hz, 8.1 Hz, 1H), 3.76 (dd, *J* = 11.3 Hz, 3.9 Hz, 1H), 3.59 (s, 3H), 3.32 (m, 2H), 1.05–1.03 (m, 63H). ^13^C NMR (75 MHz, CDCl_3_) δ 171.9, 162.0, 143.2, 136.2, 127.6, 122.9, 122.2, 119.7, 118.8, 111.2, 110.3, 104.6, 82.1, 69.9, 65.8, 61.3, 53.0, 52.3, 18.2–18.0 (18C), 12.6 (3C), 12.4 (3C), 12.0 (3C). IR (ν, cm^–1^) = 3295, 2846, 2771, 1687, 1601, 1460, 1411, 1022, 855, 730. HRMS (ESI- TOF) *m*/*z*: [M + Na]^+^ calc. for C_46_H_82_N_2_O_7_Si_3_ 881.5327; found 881.5332.

#### Methyl ((2*R*,3*R*,4*R*)-3,4-Bis­((triisopropylsilyl)­oxy)-2-(((triisopropylsilyl)­oxy)­methyl)-3,4-dihydro-2*H*-pyran-6-carbonyl)-l-valinate (**3e**)

The product was obtained as a pale-yellow oil (27%^a^–48%^b^). [α]_D_^20^ = −12 (*c* = 0.1 in CHCl_3_). ^1^H NMR (300 MHz, CDCl_3_) 7.09 (d, *J* = 9.0 Hz, 1H), 6.05 (dd, *J* = 5.3 Hz, 1.5 Hz, 1H), 4.59 (dd, *J* = 9.0 Hz, 5.4 Hz, 1H), 4.37–4.35 (m, 1H), 4.44–4.41 (m, 1H), 4.12 – 4.08 (m, 2H), 4.05 (dd, *J* = 11.5 Hz, 8.2 Hz, 1H), 3.77 (dd, *J* = 11.5 Hz, 3.6 Hz, 1H), 3.71 (s, 3H), 2.24–2.13 (m, 1H), 1.05–1.03 (m, 63H), 0.91 (t, *J* = 6.6 Hz, 6H). ^13^C NMR (75 MHz, CDCl_3_) δ 171.8, 162.2, 143.2, 136.2, 104.6, 82.3, 70.0, 65.8, 61.4, 57.2, 52.0, 31.6, 19.03, 18.2–18.1 (18C), 17.8, 12.6 (3C), 12.4 (3C), 12.0 (3C). IR (ν, cm^–1^) = 3310, 2859, 2771, 1672, 1637, 1601, 1460, 1413, 1229, 1084, 1032, 855, 730. HRMS (ESI- TOF) *m*/*z*: [M + Na]^+^ calc. for C_40_H_81_NO_7_Si_3_ 794.5218; found 794.5211.

#### Methyl ((2*R*,3*R*,4*R*)-3,4-Bis­((triisopropylsilyl)­oxy)-2-(((triisopropylsilyl)­oxy)­methyl)-3,4-dihydro-2*H*-pyran-6-carbonyl)-l-isoleucinate (**3f**)

The product was obtained as a white oil (25%^a^–52%^b^). [α]_D_^20^ = −10 (*c* = 0.1 in CHCl_3_). ^1^H NMR (300 MHz, CDCl_3_) 7.12 (d, *J* = 8.8 Hz, 1H), 6.05 (dd, *J* = 5.4 Hz, 1.5 Hz, 1H), 4.61 (dd, *J* = 8.8 Hz, 5.5 Hz, 1H), 4.44–4.40 (m, 1H), 4.12–4.07 (m, 2H), 4.12–4.07 (m, 2H), 4.05 (dd, *J* = 11.5 Hz, 8.3 Hz, 1H), 3.77 (dd, *J* = 11.5 Hz, 3.5 Hz, 1H), 3.71 (s, 3H), 1.96–1.87 (m, 1H), 1.49–1.40 (m, 2H), 1.06–1.03 (m, 63H), 0.93–0.87 (m, 6H). ^13^C NMR (75 MHz, CDCl_3_) δ 171.8, 162.1, 143.2, 104.5, 82.3, 70.0, 65.8, 61.4, 56.5, 52.0, 38.1, 25.2, 18.2–18.0 (18C), 15.4, 12.6 (3C), 12.4 (3C), 12.0 (3C), 11.5. IR (ν, cm^–1^) = 3302, 2846, 2771, 1689, 1637, 1601, 1460, 1413, 1391, 1059, 1026, 855, 730. HRMS (ESI- TOF) *m*/*z*: [M + Na]^+^ calc. for C_41_H_83_NO_7_Si_3_ 808.5375; found 808.5376.

#### Methyl ((2*R*,3*R*,4*R*)-3,4-Bis­((triisopropylsilyl)­oxy)-2-(((triisopropylsilyl)­oxy)­methyl)-3,4-dihydro-2*H*-pyran-6-carbonyl)-l-methioninate (**3g**)

The product was obtained as a pale-yellow oil (50%^a^–38%^b^). [α]_D_^20^ = −4 (*c* = 0.1 in CHCl_3_). ^1^H NMR (300 MHz, CDCl_3_) δ 7.20 (d, *J* = 8.0 Hz, 1H), 6.04 (dd, *J* = 5.3 Hz, 1.5 Hz, 1H), 4.76 (td, *J* = 11.0 Hz, 5.3 Hz, 1H), 4.43–4.39 (m, 1H), 4.12–4.07 (m, 2H), 4.03 (dd, *J* = 11.4 Hz, 8.2 Hz, 1H), 3.76 (dd, *J* = 11.5 Hz, 3.7 Hz, 1H), 3.73 (s, 3H), 2.50–2.45 (m, 2H), 2.26–2.15 (m, 1H), 2.06 (s, 3H), 2.03–1.93 (m, 1H), 1.05–1.04 (m, 63H). ^13^C NMR (75 MHz, CDCl_3_) δ 171.8, 162.1, 143.0, 104.7, 82.3, 69.9, 65.7, 61.4, 52.5, 51.5, 32.1, 29.9, 18.2–18.0 (18C), 15.5, 12.6 (3C), 12.4 (3C), 12.1 (3C). IR (ν, cm^–1^) = 3308, 2844, 2771, 1672, 1631, 1601, 1460, 1413, 1231, 1061, 1030, 855, 730. HRMS (ESI- TOF) *m*/*z*: [M + Na]^+^ calc. for C_40_H_81_NO_7_SSi_3_ 826.4939; found 826.4937.

#### Dimethyl ((2*R*,3*R*,4*R*)-3,4-Bis­((triisopropylsilyl)­oxy)-2-(((triisopropylsilyl)­oxy)­methyl)-3,4-dihydro-2*H*-pyran-6-carbonyl)-l-glutamate (3h)

The product was obtained as a brown oil (28%^a^–34%^b^). [α]_D_^20^ = −12 (c = 0.1 in CHCl_3_). ^1^H NMR (300 MHz, CDCl_3_) 7.18 (d, *J* = 7.9 Hz, 1H), 6.05 (dd, *J* = 5.3 Hz, 1.3 Hz, 1H), 4.68 (td, *J* = 7.9 Hz, 5.0 Hz, 1H), 4.43–4.40 (m, 1H), 4.11–4.00 (m, 3H), 3.79–3.74 (m, 4H), 3.66 (s, 3H), 2.40–2.23 (m, 3H), 2.08–1.96 (m, 1H), 1.05 (m, 63H). ^13^C NMR (75 MHz, CDCl_3_) δ 173.0, 171.7, 162.2, 143.0, 104.8, 82.2, 69.9, 65.7, 61.3, 52.5, 51.8, 51.5, 29.9, 27.7, 18.2–18.0 (18C), 12.6 (3C), 12.4 (3C), 12.1 (3C). IR (ν, cm^–1^) = 3297, 2846, 2771, 1687, 1635, 1601, 1413, 1391, 1026, 855, 730. HRMS (ESI-TOF) *m*/*z*: [M + K]^+^ calc. for C_41_H_81_NO_9_Si_3_ 838.5116; found 838.5112.

#### Methyl ((2*R*,3*R*,4*R*)-3,4-Bis­((triisopropylsilyl)­oxy)-2-(((triisopropylsilyl)­oxy)­methyl)-3,4-dihydro-2*H*-pyran-6-carbonyl)-l-alaninate (3i)

The product was obtained as a transparent oil (30%^a^–52%^b^). [α]_D_^20^ = −10 (*c* = 0.1 in CHCl_3_). ^1^H NMR (300 MHz, CDCl_3_) 7.13 (d, *J* = 7.3 Hz, 1H), 6.04 (dd, *J* = 5.3 Hz, 1.6 Hz, 1H), 4.62 (quin, *J* = 7.1 Hz, 1H), 4.43–4.39 (m, 1H), 4.13–4.101 (m, 3H), 3.79–3.73 (m, 4H), 1.43 (d, *J* = 5.3 Hz, 3H), 1.05 (m, 63H). ^13^C NMR (75 MHz, CDCl_3_) δ 172.9, 161.9, 143.2, 104.5, 82.2, 70.0, 65.8, 61.4, 52.4, 48.1, 18.2–18.0 (18C), 17.8, 12.6 (3C), 12.4 (3C), 12.1 (3C). IR (ν, cm^–1^) = 3306, 2846, 2771, 1691, 1601, 1460, 1413, 1059, 1026, 855, 730. HRMS (ESI-TOF) *m*/*z*: [M + Na]^+^ calc. for C_38_H_77_NO_7_Si_3_ 766.4905; found 766.4903.

#### Methyl ((2*R*,3*R*,4*R*)-3,4-Bis­((triisopropylsilyl)­oxy)-2-(((triisopropylsilyl)­oxy)­methyl)-3,4-dihydro-2*H*-pyran-6-carbonyl)­glycinate (**3j**)

The product was obtained as a white solid (38%^a^–45%^b^). mp 63.2 −63.8 °C. [α]_D_^20^ = −11 (*c* = 0.1 in CHCl_3_). ^1^H NMR (300 MHz, CDCl_3_) 7.17 (d, *J* = 5.2 Hz, 1H), 6.04 (dd, *J* = 5.2 Hz, 1.4 Hz, 1H), 4.42–4.39 (m, 1H), 4.22–4.14 (m, 1H), 4.10 – 4.00 (m, 4H), 3.77–3.72 (m, 4H), 1.04–1.02 (m, 63H). ^13^C NMR (75 MHz, CDCl_3_) δ 168.8, 162.6, 143.0, 104.7, 82.1, 69.9, 65.7, 61.3, 52.4, 41.2, 18.2–18.0 (18C), 12.6 (3C), 12.4 (3C), 12.1 (3C). IR (ν, cm^–1^) = 3298, 2842, 2769, 1696, 1601, 1475, 1413, 1033, 855, 763, 734. HRMS (ESI- TOF) *m*/*z*: [M + Na]^+^ calc. for C_37_H_75_NO_7_Si_3_ 752.4749; found 752.4745.

#### Methyl ((2*R*,3*R*,4*R*)-3,4-Bis­((triisopropylsilyl)­oxy)-2-(((triisopropylsilyl)­oxy)­methyl)-3,4-dihydro-2*H*-pyran-6-carbonyl)­glycyl-l-phenylalaninate (**3k**)

The product was obtained as a transparent oil (51%^a^). [α]_D_^20^ = +10 (*c* = 0.1 in CHCl_3_). ^1^H NMR (300 MHz, CDCl_3_) δ 7.27–7.21 (m, 4H), 7.09 (d, *J* = 7.9 Hz, 2H), 6.54 (d, *J* = 7.9 Hz, 1H), 6.09 (dd, *J* = 5.2 Hz, 1.3 Hz, 1H), 4.87–4.80 (m, 1H), 4.41–4.40 (m, 1H), 4.14–4.09 (m, 2H), 4.053.98 (m, 2H), 3.89–3.76 (m, 2H), 3.69 (s, 3H), 3.13 (dd, *J* = 13.8 Hz, 6.0 Hz, 1H), 3.06 (dd, *J* = 13.8 Hz, 6.1 Hz, 1H), 1.06–1.03 (m, 63H). ^13^C NMR (75 MHz, CDCl_3_) δ 171.6, 168.2, 163.1, 142.9, 135.8, 129.3 (2C), 128.7 (2C), 127.3, 105.1, 82.0, 69.9, 65.8, 61.2, 53.5, 52.4, 43.4, 38.0, 18.2–18.0 (18C), 12.5 (3C), 12.4 (3C), 12.1 (3C). IR (ν, cm^–1^) = 3298, 3196, 2846, 2771, 1689, 1618, 1596, 1460, 1413, 1026, 855, 730. HRMS (ESI- TOF) *m*/*z*: [M + Na]^+^ calc. for C_46_H_84_N_2_O_8_Si_3_ 899.5433; found 899.5431.

#### Methyl *N*,*S*-bis­((2*R*,3*R*,4*R*)-3,4-Bis­((triisopropylsilyl)­oxy)-2-(((triisopropylsilyl)­oxy)­methyl)-3,4-dihydro-2*H*-pyran-6-carbonyl)-l-cysteinate (3l)

The product was obtained as a pale-yellow oil (96%^a^). [α]_D_^20^ = +8 (c = 0.1 in CHCl_3_). ^1^H NMR (300 MHz, CDCl_3_) 7.32 (d, *J* = 7.6 Hz, 1H), 6.04 (dd, *J* = 5.2 Hz, 1.5 Hz, 1H), 5.88 (d, *J* = 5.3 Hz, 1.4 Hz, 1H), 4.83–4.76 (m, 1H), 4.42–4.40 (m, 2H), 4.17 (m, 1H), 4.14–4.09 (m, 3H), 4.01 (dd, *J* = 11.4 Hz, 8.0 Hz, 1H), 3.93–3.89 (m, 2H), 3.79 (dd, *J* = 11.4 Hz, 4.0 Hz, 1H), 3.69 (s, 3H), 3.46 (dd, *J* = 13.9 Hz, 5.2 Hz, 1H), 3.35 (dd, *J* = 13.9 Hz, 6.3 Hz, 1H), 1.05–1.03 (m, 126H). ^13^C NMR (75 MHz, CDCl_3_) δ 188.6, 170.1, 162.0, 146.7, 143.1, 104.9, 103.8, 82.1, 81.9, 69.9, 69.8, 66.0, 65.9, 61.3, 61.2, 52.6, 52.1, 30.2, 18.2–18.0 (36C), 12.67 (3C), 12.65 (3C), 12.47 (3C), 12.6 (3C), 12.16 (3C), 12.13 (3C). IR (ν, cm^–1^) = 3300, 2846, 2771, 1695, 1631, 1601, 1460, 1413, 1059, 1028, 855, 730. MALDI (TOF/TOF) *m*/*z*: [M + Na]^+^ calc. for C_72_H_145_NO_12_SSi_6_ 1438.9000; found 1438.9758.

#### 4-((*S*)-2-((2*R*,3*R*,4*R*)-3,4-Bis­((triisopropylsilyl)­oxy)-2-(((triisopropylsilyl)­oxy)­methyl)-3,4-dihydro-2*H*-pyran-6-carboxamido)-3-methoxy-3-oxopropyl)­phenyl (2*R*,3*R*,4*R*)-3,4-bis­((triisopropylsilyl)­oxy)-2-(((triisopropylsilyl)­oxy)­methyl)-3,4-dihydro-2*H*-pyran-6-carboxylate (**3m**)

The product was obtained as a pale-yellow oil (13%^a^). [α]_D_^20^ = −8 (*c* = 0.1 in CHCl_3_). ^1^H NMR (300 MHz, CDCl_3_) 7.17 (d, *J* = 7.7 Hz, 1H), 7.13 (d, *J* = 8.3 Hz, 2H), 7.02 (d, *J* = 8.3 Hz, 2H), 6.22 (d, *J* = 5.4 Hz, 1H), 6.06 (d, *J* = 5.4 Hz, 1H), 4.94–4.87 (m, 1H), 4.53–4.48 (m, 1H), 4.40–4.38 (m, 1H), 4.22 (m, 1H), 4.19–4.17 (m, 1H), 4.12–4.10 (m, 1H), 4.07–4.05 (m, 1H), 4.03–3.95 (m, 3H), 3.76 (dd, *J* = 11.5 Hz, 3.9 Hz, 1H), 3.65 (s, 3H), 3.14–3.11 (m, 2H), 1.09–1.02 (m, 126H). ^13^C NMR (75 MHz, CDCl_3_) δ 171.2, 161.9, 161.4, 149.8, 143.1, 142.0, 133.7, 130.3 (2C), 121.7 (2C), 109.6, 104.8, 82.2, 81.7, 69.9, 69.5, 66.0, 65.8, 61.3, 53.5, 52.3, 37.8, 18.2–18.0 (36C), 12.67 (3C), 12.62 (3C), 12.5 (3C), 12.4 (3C), 12.18 (3C), 12.11 (3C). IR (ν, cm^–1^) = 2831, 2771, 1670, 1460, 1413, 1229, 1084, 1035, 855. 724. MALDI (TOF/TOF) *m*/*z*: [M + Na]^+^ calc. for C_78_H_149_NO_13_Si_6_ 1498.9542; found 1499.0957.

#### Methyl *N*2,*N*6-Bis­((2*R*,3*R*,4*R*)-3,4-Bis­((triisopropylsilyl)­oxy)-2-(((triisopropylsilyl)­oxy)­methyl)-3,4-dihydro-2*H*-pyran-6-carbonyl)-l-lysinate (**3n**)

The product was obtained as a pale-yellow oil (5%^a^–15%^b^). [α]_D_^20^ = −7 (c = 0.1 in CHCl_3_). ^1^H NMR (300 MHz, CDCl_3_) 7.11 (d, *J* = 8.1 Hz, 1H), 6.70 (d, *J* = 5.8 Hz, 1H), 6.05–6.01 (m, 2H), 4.67–4.60 (m, 1H), 4.43–4.36 (m, 2H), 4.11–4.00 (m, 6H), 3.80–3.67 (m, 5H), 3.29–3.21 (m, 2H), 1.97–1.84 (m, 2H), 1.77–1.67 (m, 2H), 1.55–1.48 (m, 4H), 1.05 (m, 126H). ^13^C NMR (75 MHz, CDCl_3_) δ 172.2, 162.4, 162.1, 143.3, 143.1, 104.6, 104.0, 82.2, 82.0, 70.1, 69.9, 65.84, 65.82, 61.3, 52.4, 51.9, 39.0, 32.3, 29.3, 22.7, 18.2–17.8 (36C), 12.6–12.1 (18C). IR (ν, cm^–1^) = 3322, 2846, 2771, 1691, 1631, 1601, 1460, 1413, 1218, 1048, 1026, 855, 730. MALDI (TOF/TOF) *m*/*z*: [M + Na]^+^ calc. for C_75_H_152_N_2_O_12_Si_6_ 1463.9858; found 1464.0276.

#### Methyl (*S*)-2-((2*R*,3*R*,4*R*)-3,4-Bis­((triisopropylsilyl)­oxy)-2-(((triisopropylsilyl)­oxy) methyl)-3,4-dihydro-2*H*-pyran-6-carboxamido)-3-(4-hydroxy-3-((*E*)-styryl)­phenyl) propanoate (**3o**)

The product was obtained as a beige oil (24%^a^). [α]_D_^20^ = +11 (*c* = 0.1 in CHCl_3_). ^1^H NMR (300 MHz, CDCl_3_) 7.53–7.50 (m, 2H), 7.36–7.24 (m, 5H), 7.18 (d, *J* = 7.71 Hz, 1H), 7.07 (d, *J* = 16 Hz, 1H), 6.89 (dd, *J* = 8.2 Hz, 2.1 Hz, 1H), 6.80 (d, *J* = 8.2 Hz, 1H), 6.06 (dd, *J* = 5.3 Hz, 1.7 Hz, 1H), 5.43 (br, 1H), 4.90–4.83 (m, 1H), 4.40–4.36 (m, 1H), 4.13–3.99 (m, 3H), 3.75 (dd, *J* = 11.5 Hz, 3.7 Hz, 1H), 3.68 (s, 3H), 3.09–3.06 (m, 2H), 1.05–1.00 (m, 63H). ^13^C NMR (75 MHz, CDCl_3_) δ 171.1, 162.1, 152.5, 143.1, 137.8, 130.2, 129.4, 128.7 (2C), 128.4, 127.9, 127.6, 126.7 (2C), 124.9, 123.2, 116.2, 104.8, 82.2, 70.0, 65.8, 61.4, 53.8, 52.3, 37.8, 18.2–18.1 (18C), 12.6 (3C), 12.4 (3C), 12.1 (3C). IR (ν, cm^–1^) = 3302, 2861, 2771, 1680, 1600, 1411, 1333, 1218, 1052, 1028, 981, 966, 855, 732. HRMS (ESI-TOF) *m*/*z*: [M + Na]^+^ calc. for C_52_H_87_NO_8_Si_3_ 960.5637; found 960.5625.

#### Methyl (*S*)-2-((2*R*,3*R*,4*R*)-3,4-Bis­((triisopropylsilyl)­oxy)-2-(((triisopropylsilyl)­oxy)­methyl)-3,4-dihydro-2*H*-pyran-6-carboxamido)-3-(3-((*E*)-4-fluorostyryl)-4-hydroxyphenyl)­propanoate (**3p**)

The product was obtained as a beige oil (27%^a^). [α]_D_^20^ = +14 (*c* = 0.1 in CHCl_3_). ^1^H NMR (300 MHz, CDCl_3_) δ 7.49–7.45 (m, 2H), 7.19–7.16 (m, 2H), 7.06–6.99 (m, 3H), 6.89 (dd, *J* = 8.4 Hz, 2.0 Hz, 1H), 6.68 (d, *J* = 8.1 Hz, 1H), 6.06 (dd, *J* = 5.4 Hz, 1.6 Hz, 1H), 5.41 (br, 1H), 4.90–4.83 (m, 1H), 4.39–4.37 (m, 1H), 4.10–3.99 (m, 3H), 3.75 (dd, *J* = 11.5 Hz, 3.5 Hz, 1H), 3.67 (s, 3H), 3.13–3.00 (m, 2H). 1.04–1.00 (m, 63H). ^13^C NMR (75 MHz, CDCl_3_) δ 171.6, 162.4 (d, *J* = 245 Hz, C–F), 162.1, 152.4, 143.0, 134.1 (d, *J* = 3.3 Hz, C–F), 129.4, 128.8, 128.5, 128.2 (d, *J* = 7.8 Hz, C–F), 127.8, 124.7, 123.0, 116.2, 115.7 (d, *J* = 21.5 Hz, C–F), 104.8, 82.2, 69.9, 65.8, 61.4, 53.8, 52.3, 37.8, 18.2–18.0 (18C), 12.6 (3C), 12.4 (3C), 12.1 (3C). IR (ν, cm^–1^) = 3302, 2846, 2771, 1687, 1596, 1460, 1413, 1188, 1052, 1026, 855, 728. HRMS (ESI-TOF) *m*/*z*: [M + Na]^+^ calc. for C_52_H_86_FNO_8_Si_3_ 978.5543; found 978.5542.

#### Methyl ((2*S*,4*S*)-2-Methyl-4-((triisopropylsilyl)­oxy)-3,4-dihydro-2*H*-pyran-6-carbonyl)-l-tryptophanate (**4**)

The product was obtained as a white solid (40%^a^). [α]_D_^20^ = +46 (*c* = 0.1 in CHCl_3_). mp 129.1–130.7 °C ^1^H NMR (300 MHz, CDCl_3_) δ 8.12 (br, 1H), 7.54 (d, *J* = 7.5 Hz, 1H), 7.33 (d, *J* = 8.0 Hz, 1H), 7.18–7.13 (m, 1H), 7.09–7.04 (m, 1H), 6.97 (d, *J* = 2.1 Hz, 1H), 6.08 (dd, *J* = 5.1 Hz, 1.4 Hz, 1H), 5.02–4.96 (m, 1H), 4.35–4.28 (m, 1H), 4.17–4.14 (m, 2H), 3.92–3.91 (m, 1H), 3.65 (s, 3H), 3.37 (d, *J* = 5.2 Hz, 2H), 1.25 (t, *J* = 7.0 Hz, 1H), 1.10–1.01 (m, 42H). ^13^C NMR (75 MHz, CDCl_3_) δ 172.0, 162.3, 142.7, 136.2, 127.6, 122.8, 122.2, 119.8, 118.9, 111.2, 110.4, 104.6, 75.7, 73.1, 66.6, 53.2, 52.3, 27.8, 18.3–18.1 (12C), 12.6 (3C), 12.5 (3C). IR (ν, cm^–1^) = 3159, 2844, 2771, 1687, 1596, 1475, 1411, 1296, 1091, 1030, 853, 773. HRMS (ESI-TOF) *m*/*z*: [M + H]^+^ calc. for C_37_H_62_N_2_O_6_Si_2_ 687.4224; found 687.3936.

#### Procedure for Deprotection of Glucal Amides (**5**)

A solution of TBAF (1 M in THF, 1.2 mmol, 6 equiv) was added to a solution of gluco-amide **3d** (172 mg; 0.2 mmol) in THF at room temperature. The mixture was stirred at room temperature for 5 h. The organic solvent was evaporated under vacuum, and the residue was purified by flash column chromatography using MeOH/AcOEt as the eluent (0% to 10%).

#### Methyl ((2*R*,3*S*,4*R*)-3,4-Dihydroxy-2-(hydroxymethyl)-3,4-dihydro-2*H*-pyran-6-carbonyl)-l-tryptophanate (5)

The product was obtained as a white solid (77%). [α]_D_^20^ = −29 (*c* = 0.1 in MeOH). mp 83.9–85.1 °C. ^1^H NMR (300 MHz, MeOD) δ 7.59 (d, *J* = 7.7 Hz, 1H), 7.41 (d, *J* = 8.0 Hz, 1H), 7.19–7.06 (m, 3H), 5.84 (d, *J* = 2.7 Hz, 1H), 4.84 (m, 1H), 4.26 (dd, *J* = 7.3 Hz, 2.6 Hz, 1H), 3.98–3.83 (m, 3H), 3.74 (s, 3H), 3.66–3.61 (m, 1H), 3.43 (dd, *J* = 14.6 Hz, 5.7 Hz, 1H), 3.33 (dd, *J* = 14.6 Hz, 7.5 Hz, 1H). ^13^C NMR (75 MHz, MeOD) δ 173.6, 163.5, 146.3, 138.0, 128.6, 124.4, 122.4, 119.8, 119.1, 112.3, 110.5, 109.7, 81.7, 70.4, 70.1, 62.1, 54.8, 52.7, 28.2. IR (ν, cm^–1^) = 3218, 2853, 1673, 1585, 1473, 1460, 1411, 1391, 1298, 1181, 1037, 1000, 980, 720. HRMS (ESI- TOF) *m*/*z*: [M + H] ^+^ calc. for C_19_H_22_N_2_O_7_ 391.1505; found 391.1552.

### Steady State Absorption and Fluorescence Spectra

The absorption spectrum was recorded on a Varian Cary 50 Bio UV–vis spectrophotometer using quartz cuvettes with 1.0 cm optical path length at 25 ± 1 °C. The fluorescence spectra were recorded on a FS5 spectrofluorometer using (10 mm × 10 mm) quartz cuvettes with PTFE (white) stopper at 25 ± 1 °C. The measurement of absolute fluorescence quantum yield was performed using a SC-30 integrating sphere module. The sample was diluted to an absorbance of 0.08 at the excitation wavelength. Fluorescence spectra of the blank (solvent, CHCl3) and sample were recorded under the same experimental conditions; excitation wavelength: 280 nm, scan limits: from 260 to 800 nm, excitation bandwidth: 10 nm, emission bandwidth: 1.47 nm, wavelength step size: 1 nm, integration time: 1 s (= 0.2 s dwell time accumulated 5-times), signal level: ∼10^6^ cps.

### Fluorescence Quantum Yields (Φ_f_)

Fluorescence quantum yield was determined by the absolute method in which the integration sphere measured the number of absorbed and emitted photons by a sample. The absolute fluorescence quantum yield (Φ_f_) is calculated using eq 1,

1 $$\Phi_{f}=\frac{E_{s}}{S_{b}-S_{s}}$$

where the subscripts s and b stand for sample and blank, respectively, Φ_f_ is the fluorescence quantum yield, *E* is the integrated area under the emission curve and *S* is the integrated area under the excitation scatter curve.

## Supplementary Material



## Notes

NMR spectra for all the compounds (PDF)
