# Supplementary material for: Carbonylative Coupling of 1‑Iodoglucal and Amino Acids: Access to New Fluorescent Sugar Amino Acids
Source: ACS Omega. 2025 Jul 15;10(29):32444–53. doi: 10.1021/acsomega.5c04865 (PMC12311724; doi:10.1021/acsomega.5c04865)

Carbonylative coupling of 1-iodo glucal and amino acids: Access to new fluorescent sugar amino acid

Pamela M. Silva,[a] Monica F. Z. J. Toledo,[b] Flávia Manarin[c], Daniel C. Pimenta,[d] Nicaely M. O. Pereira,[e] Erick. L. Bastos,[e] Vinicius M. Silva,[b] Milene M. Hornink,*,[b] Hélio A. Stefani*,[b]

[a]Centro Universitário São Camilo, São Paulo, SP, Brazil, [b]Departamento de Farmácia, Faculdade de Ciências Farmacêuticas, Universidade de São Paulo, São Paulo, SP, Brazil, [c]Centro de Engenharias e Ciências Exatas, Unioeste, Toledo, PR-Brasil. [d]Instituto Butantan, São Paulo, SP, Brazil, [e]Departamento de Química Fundamental, Instituto de Química, Universidade de São Paulo, São Paulo, SP, Brazil.

Corresponding Author: hstefani@usp.br, milenemacedohornink@usp.br

Table of Contents

1. NMR (^{1}H, ^{13}C) Spectra of Products..2

1. NMR (^{1}H, ^{13}C) Spectra of Products

^{1}H NMR spectra (300 MHz, $CDCl_3$) of **3a**

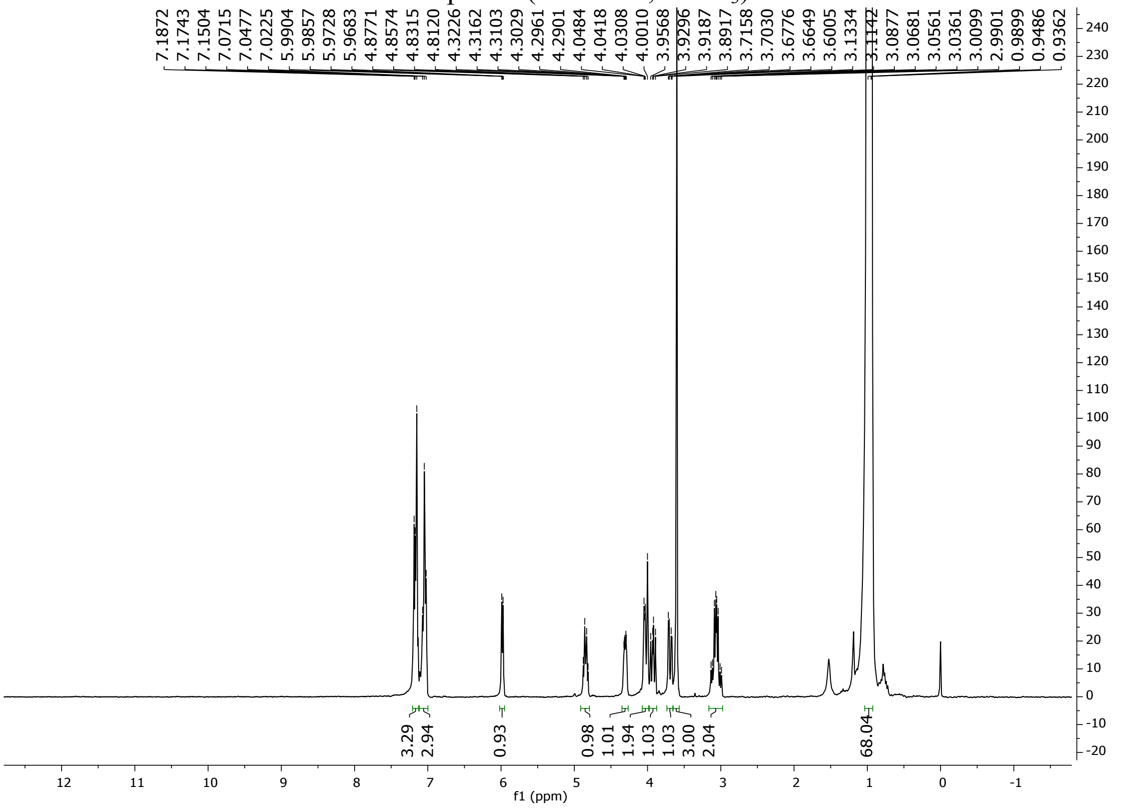

^{13}C NMR spectra (75 MHz, $CDCl_3$) of **3a**

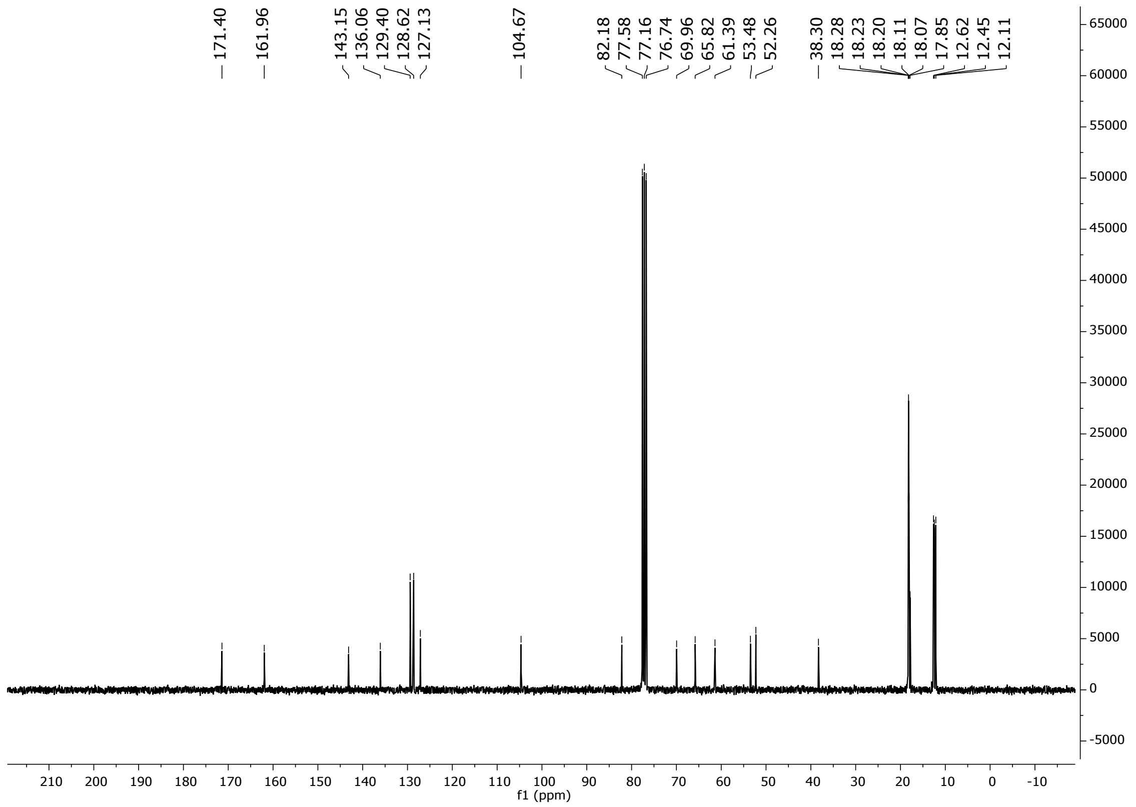

^{1}H NMR spectra (300 MHz, $CDCl_3$) of **3b**

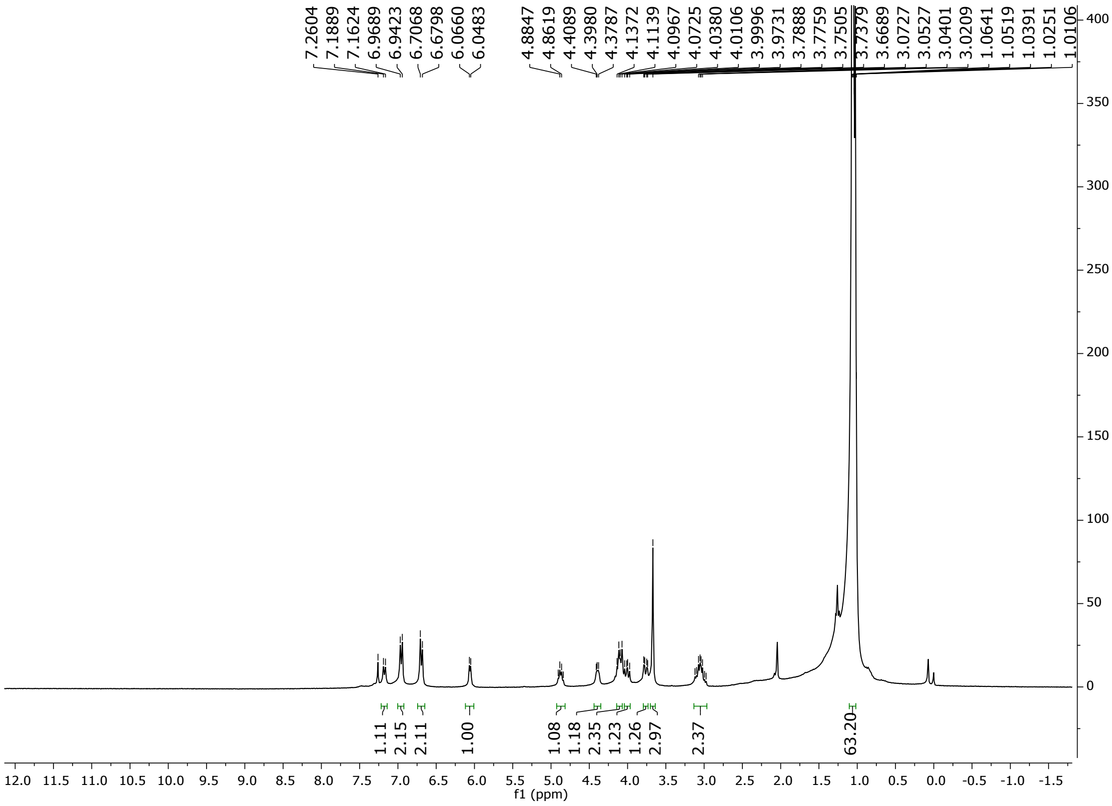

^{13}C NMR spectra (75 MHz, $CDCl_3$) of **3b**

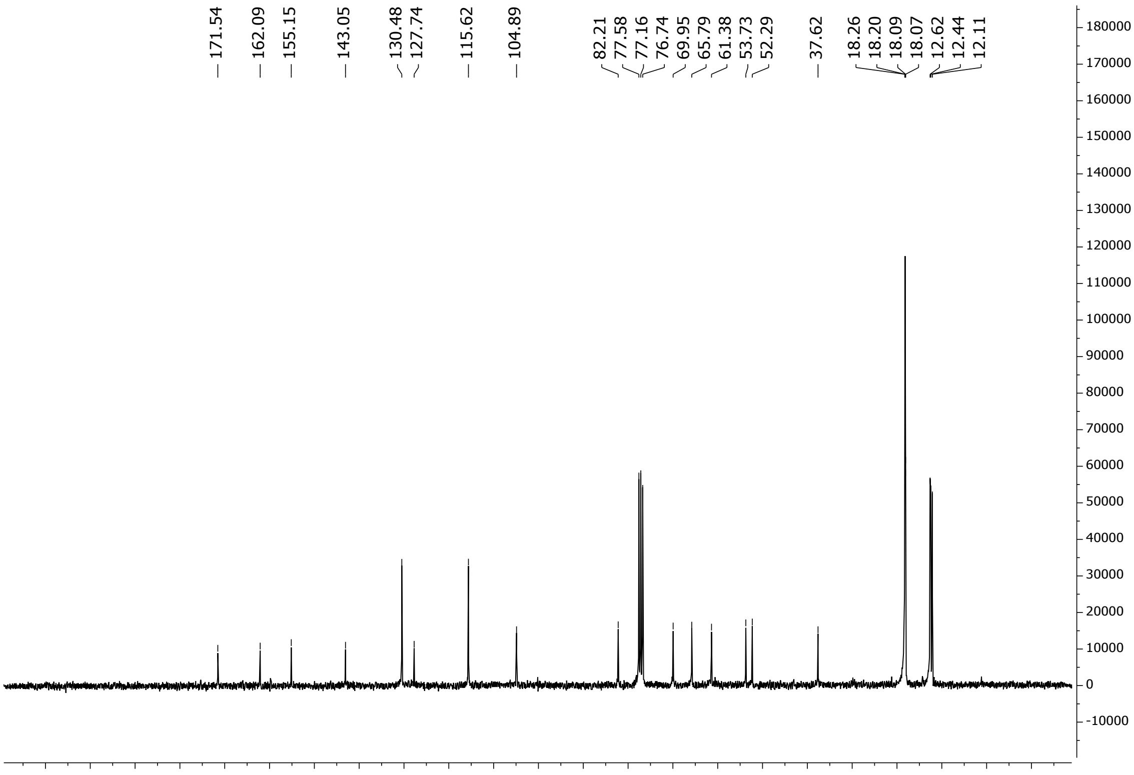

^{1}H NMR spectra (300 MHz, $CDCl_3$) of **3c**

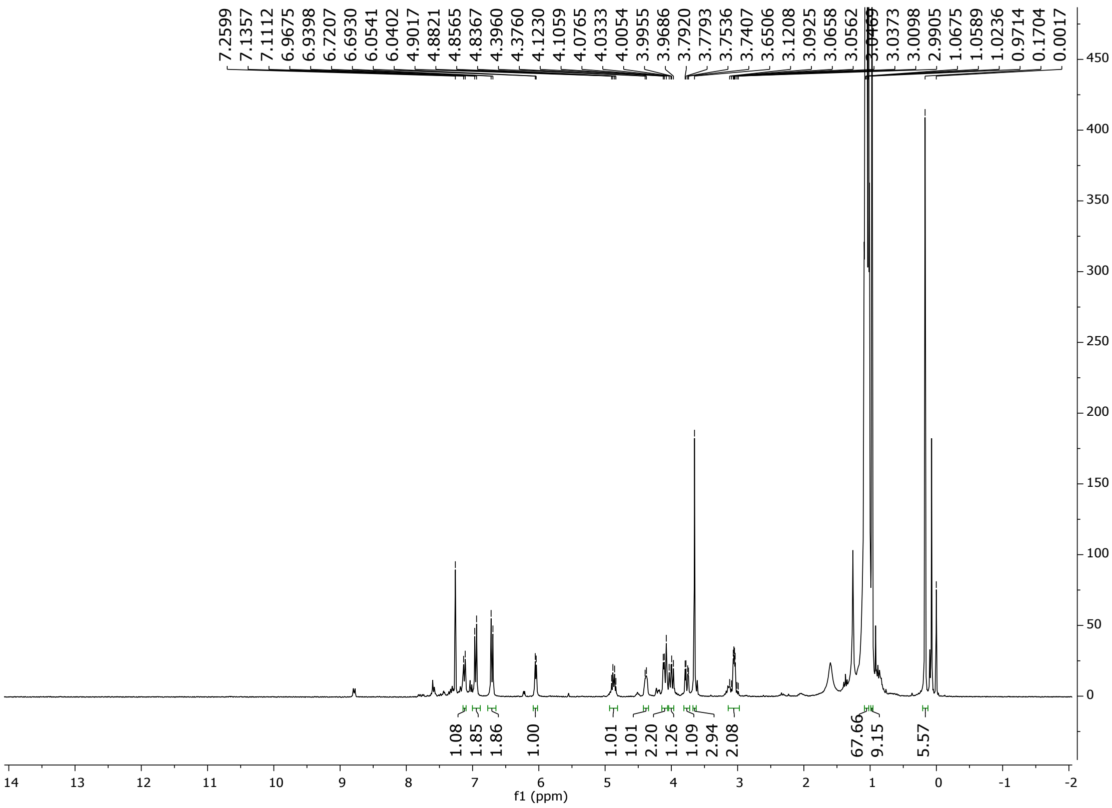

^{13}C NMR spectra (75 MHz, $CDCl_3$) of **3c**

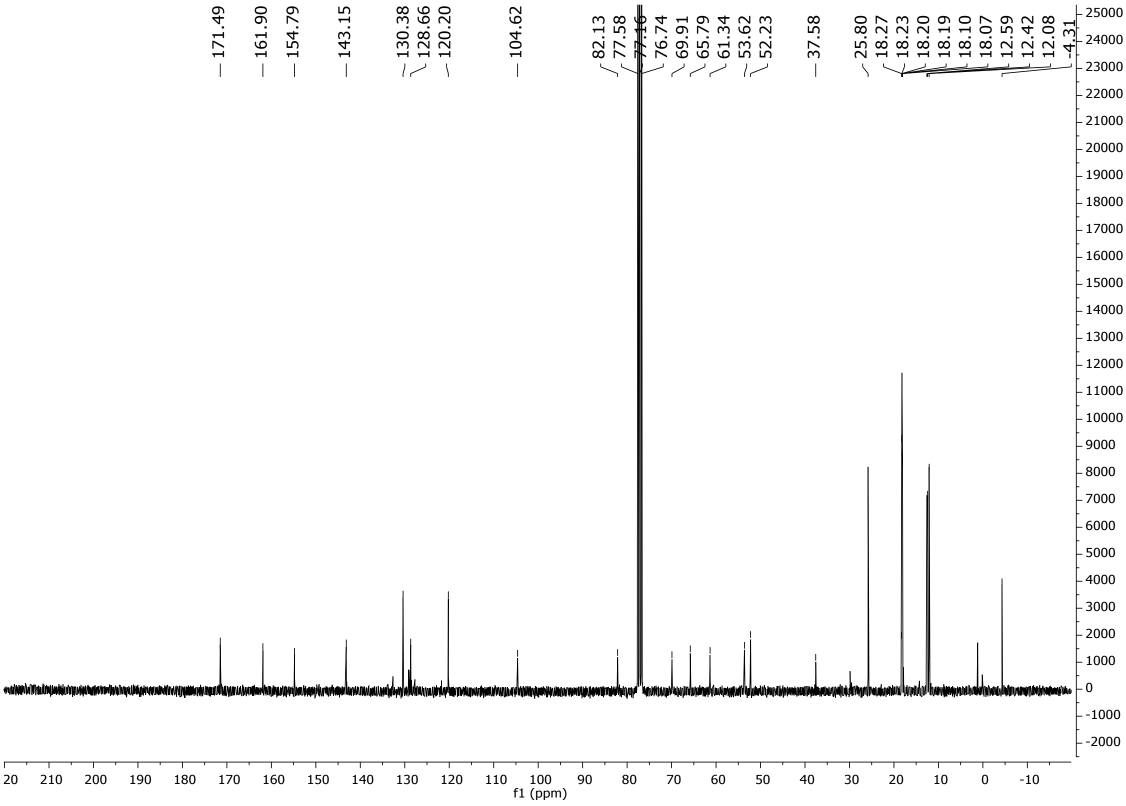

^{1}H NMR spectra (300 MHz, $CDCl_3$) of **3d**

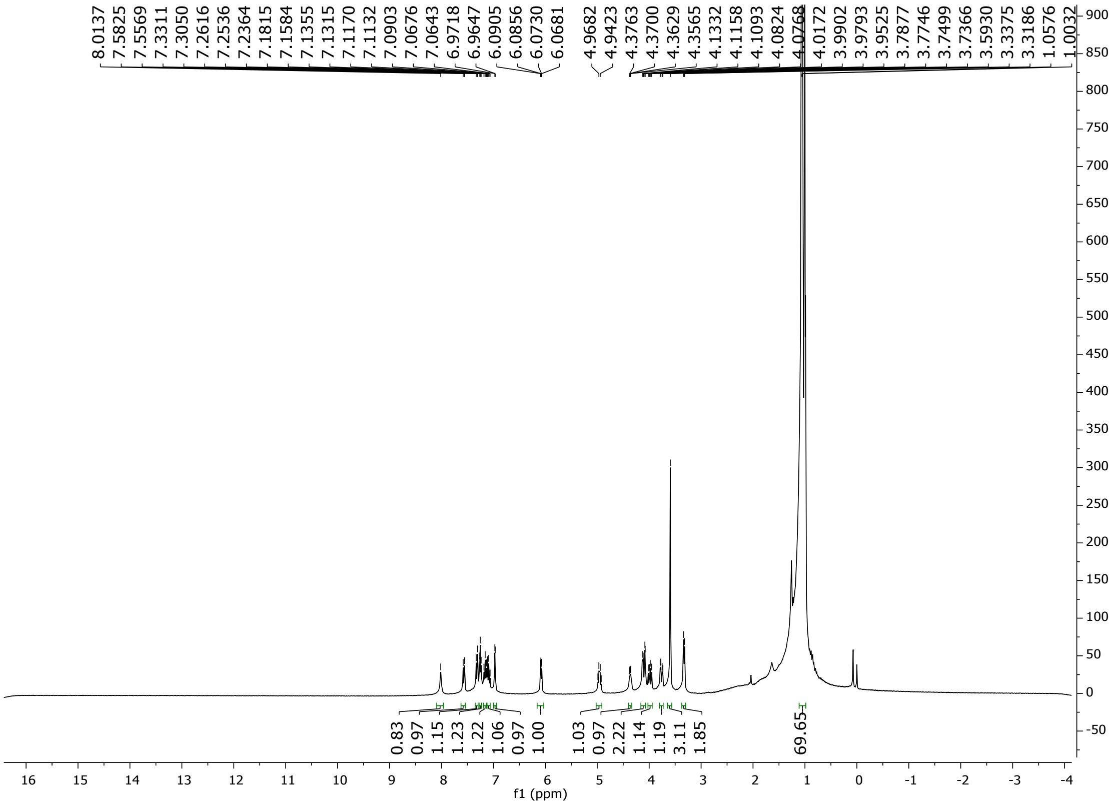

^{13}C NMR spectra (75 MHz, $CDCl_3$) of **3d**

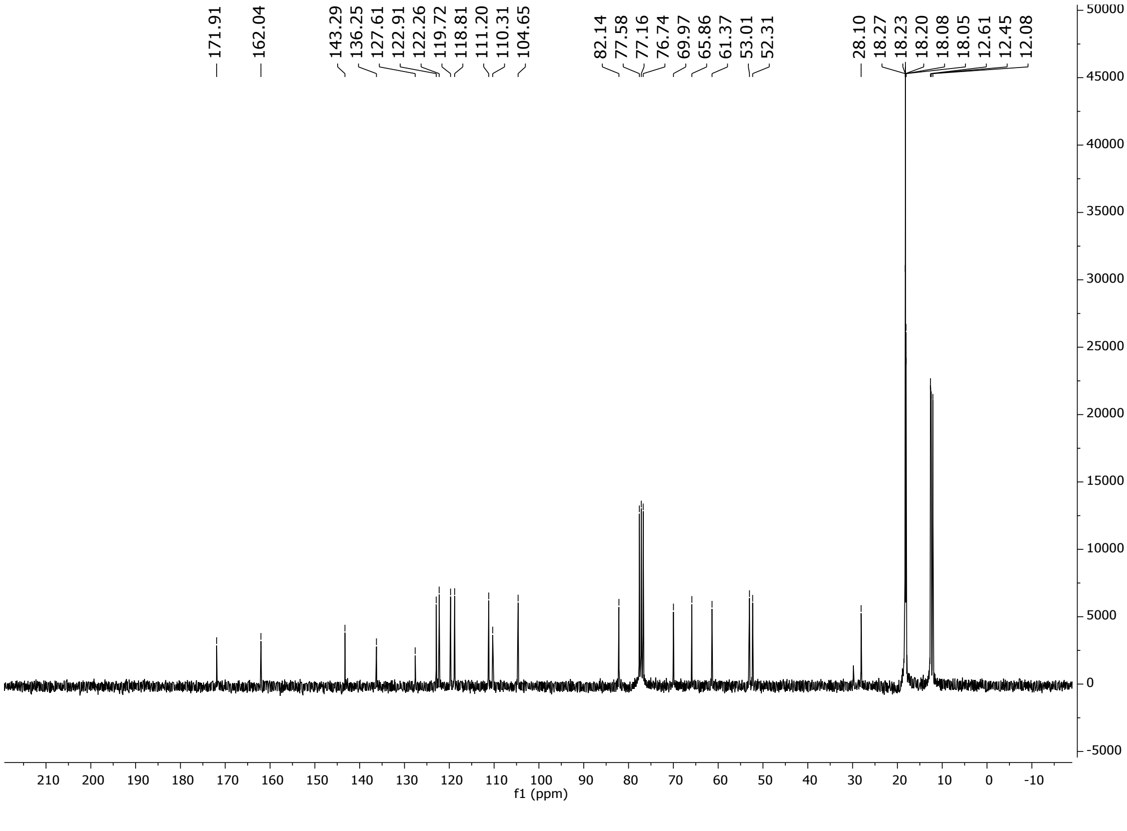

^{1}H NMR spectra (300 MHz, $CDCl_3$) of **3e**

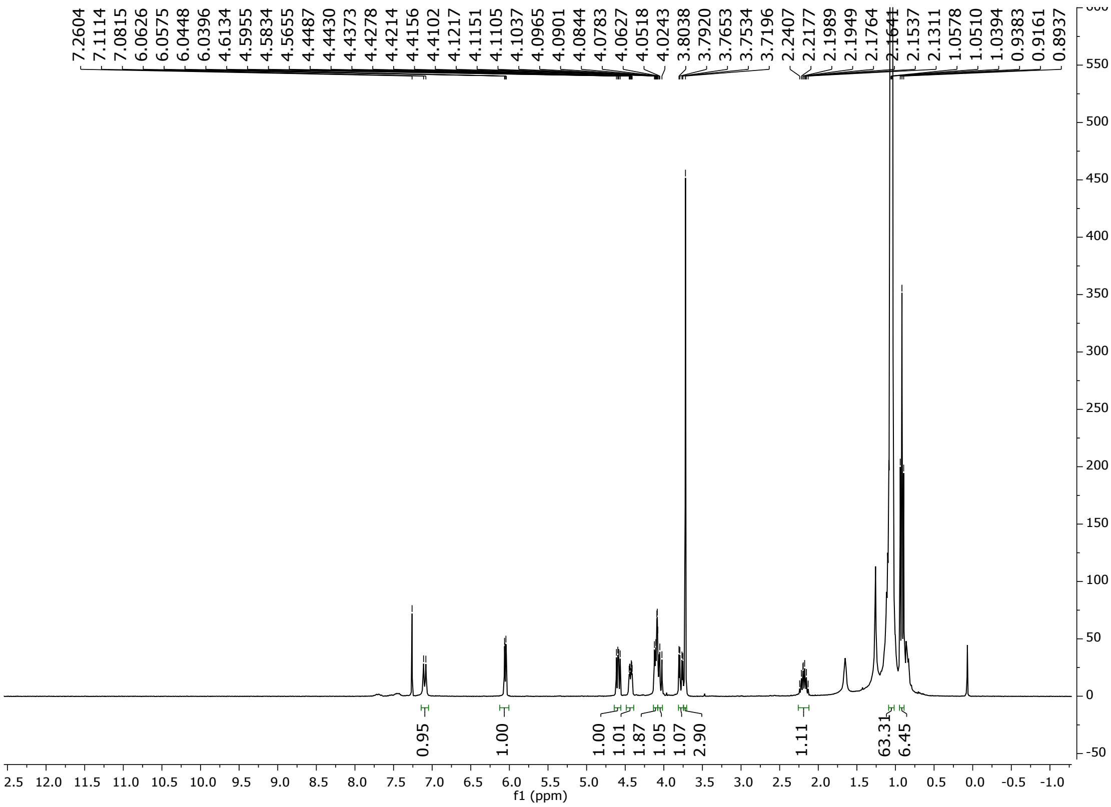

^{13}C NMR spectra (75 MHz, $CDCl_3$) of **3e**

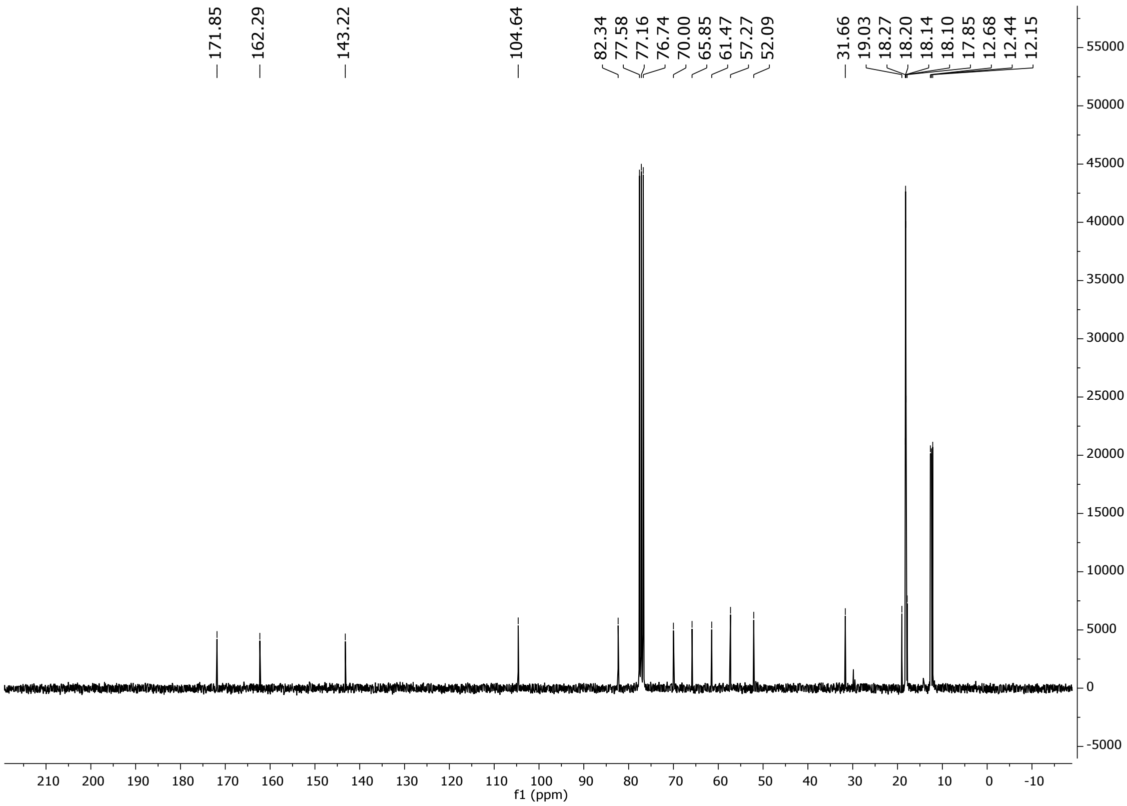

^{1}H NMR spectra (300 MHz, $CDCl_3$) of **3f**

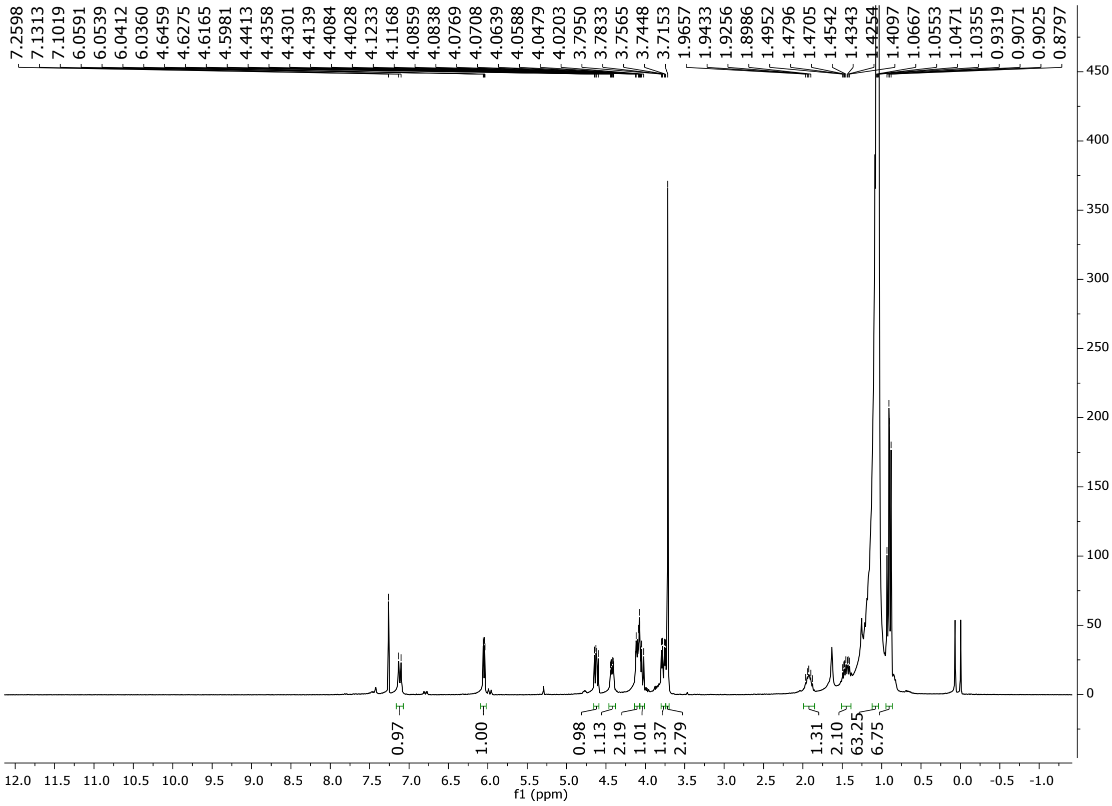

^{13}C NMR spectra (75 MHz, $CDCl_3$) of **3f**

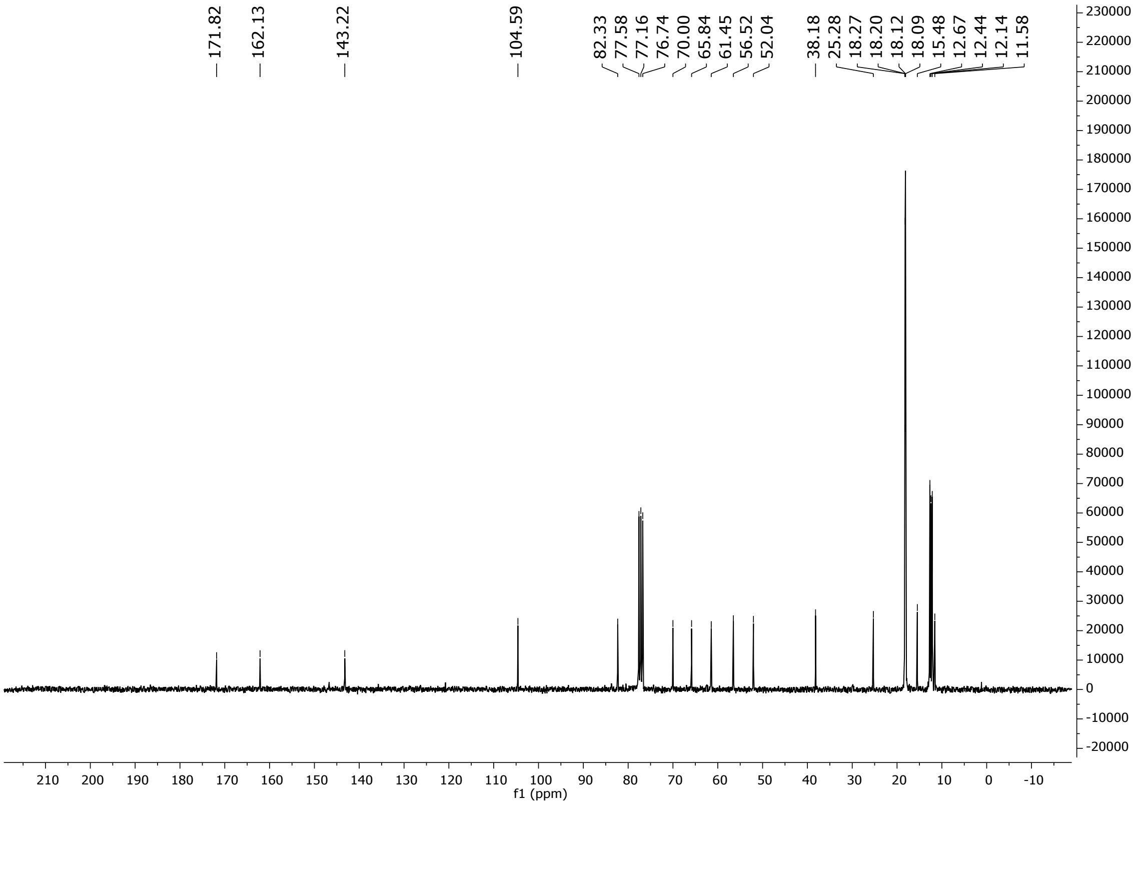

^{1}H NMR spectra (300 MHz, $CDCl_3$) of **3g**

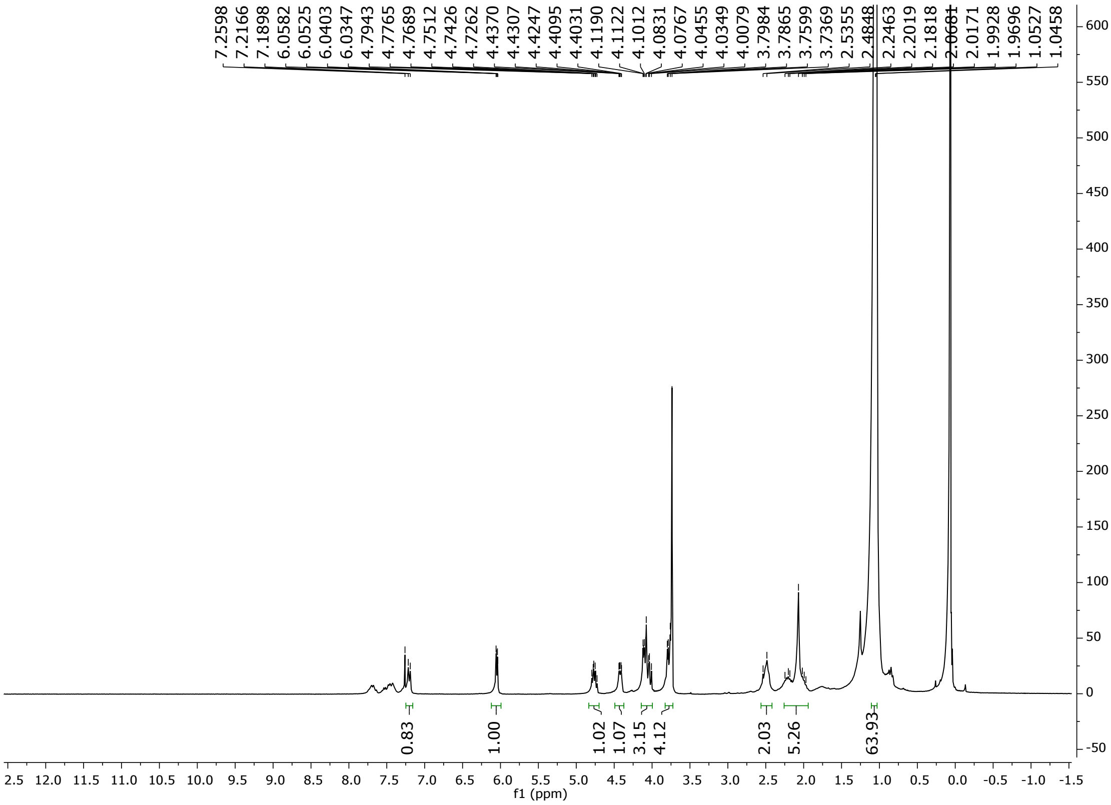

^{13}C NMR spectra (75 MHz, $CDCl_3$) of **3g**

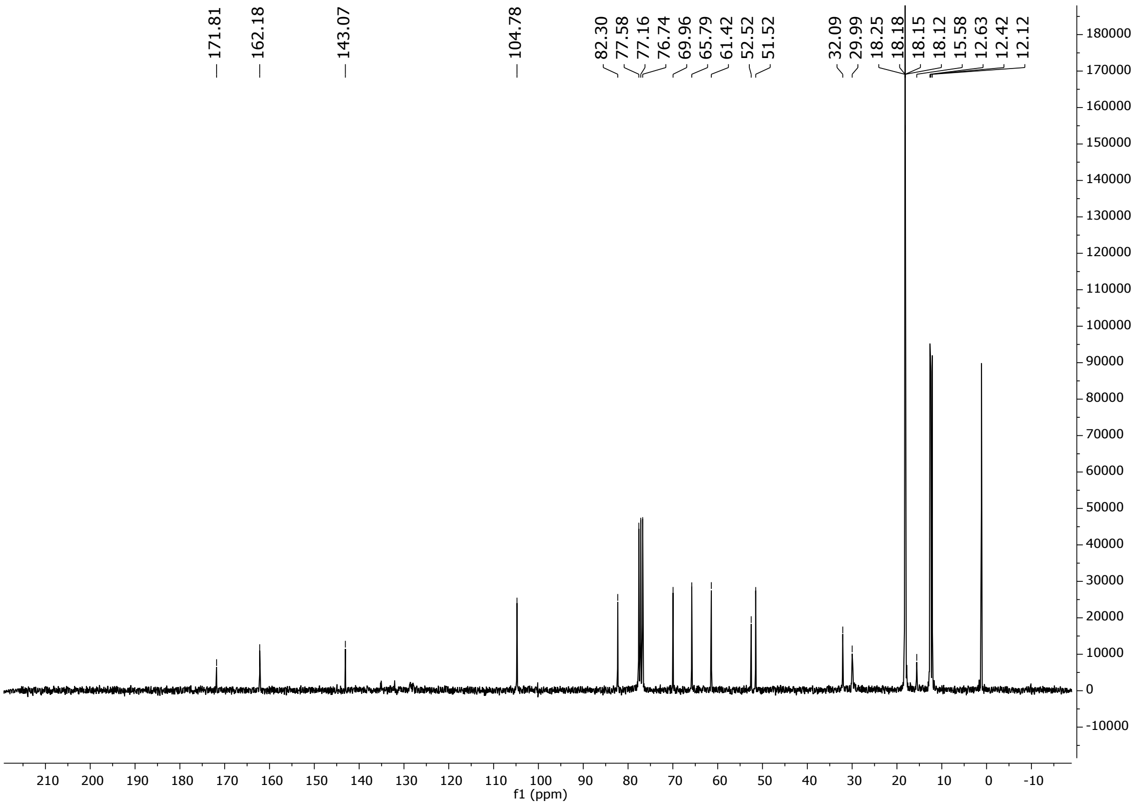

^{1}H NMR spectra (300 MHz, $CDCl_3$) of **3h**

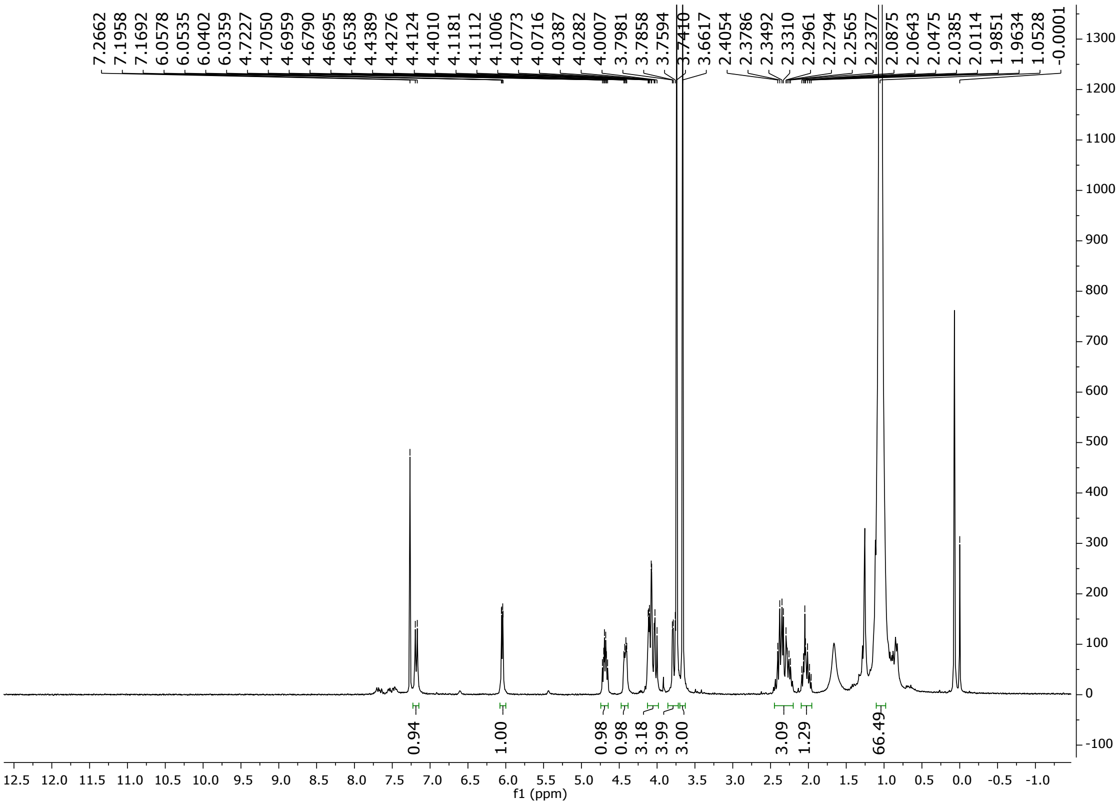

^{13}C NMR spectra (75 MHz, $CDCl_3$) of **3h**

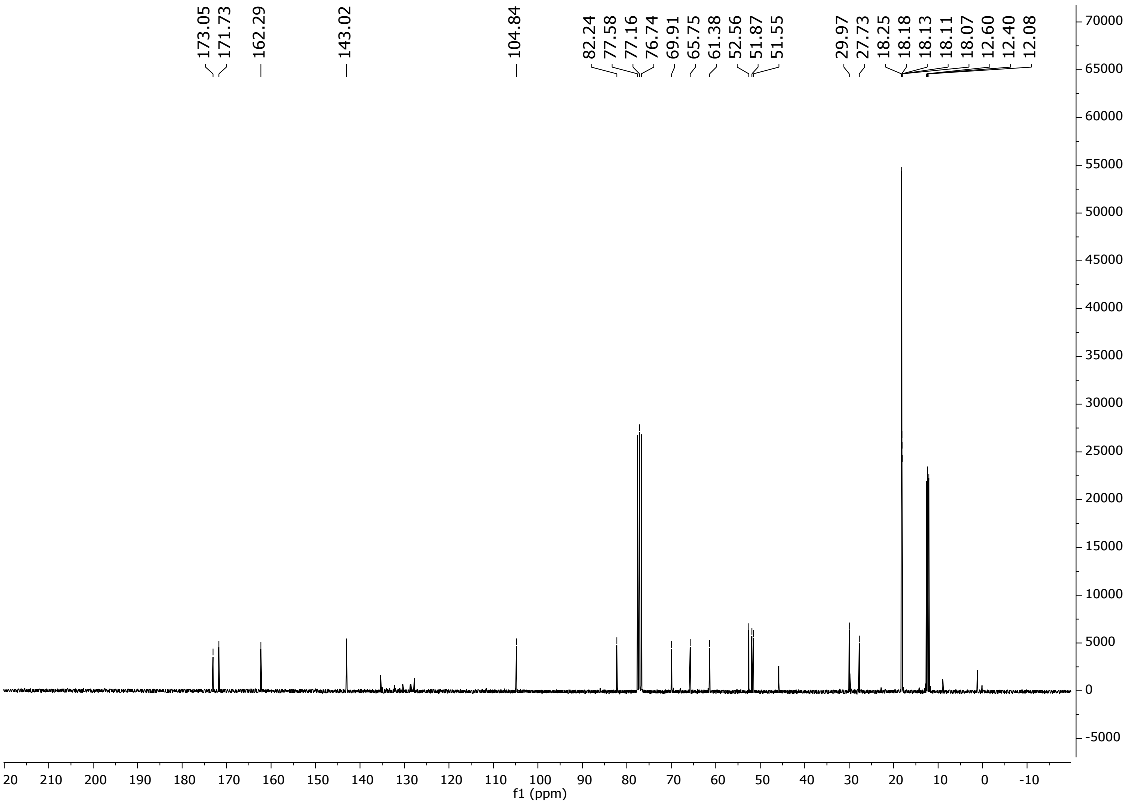

^{1}H NMR spectra (300 MHz, $CDCl_3$) of **3i**

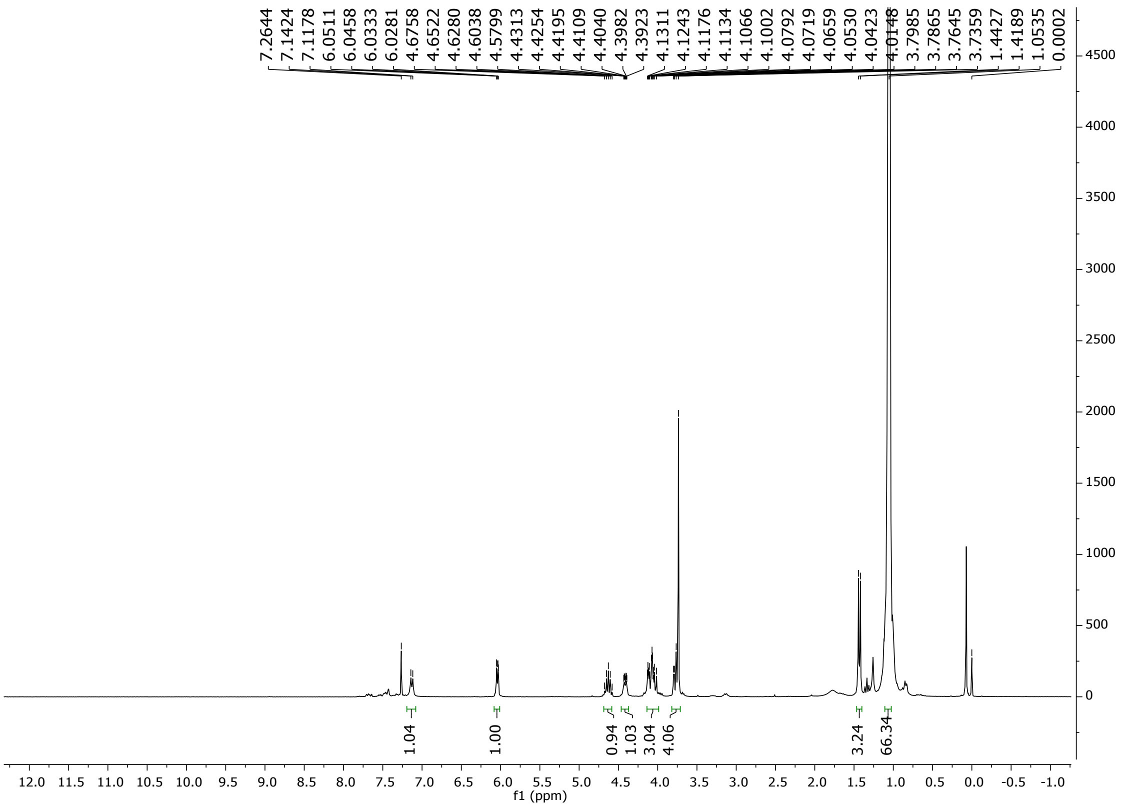

^{13}C NMR spectra (75 MHz, $CDCl_3$) of **3i**

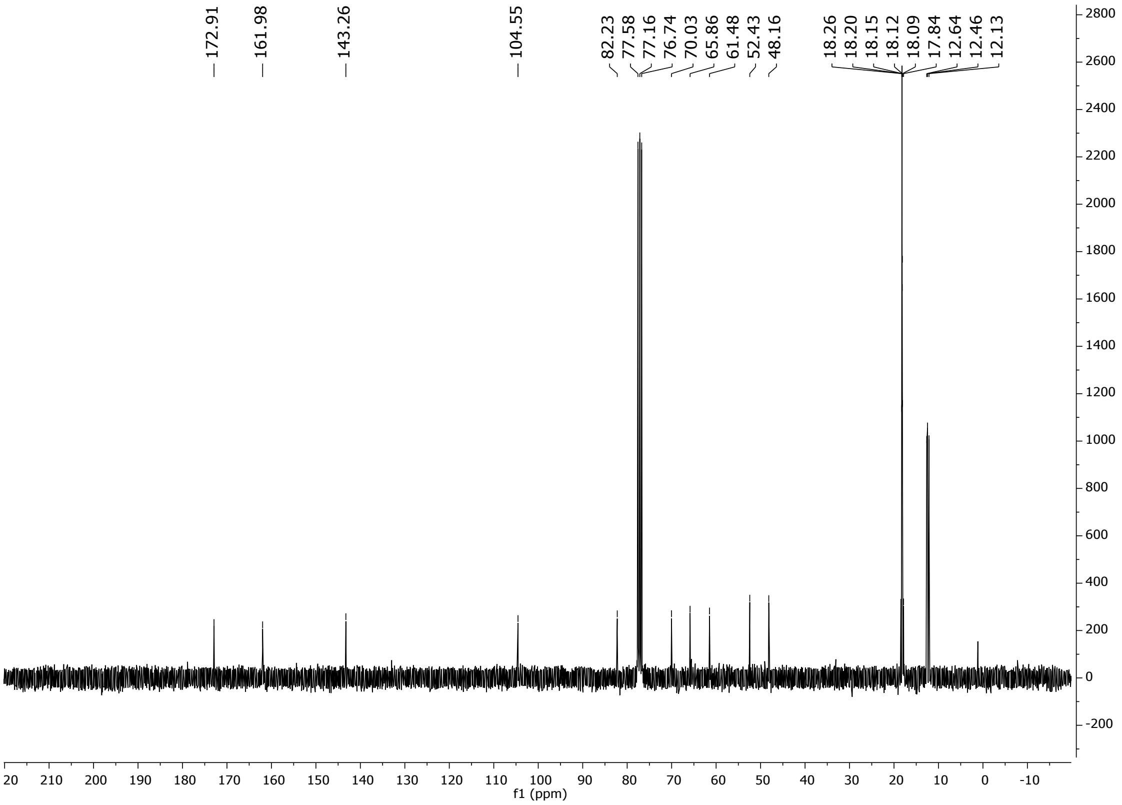

^{1}H NMR spectra (300 MHz, $CDCl_3$) of **3j**

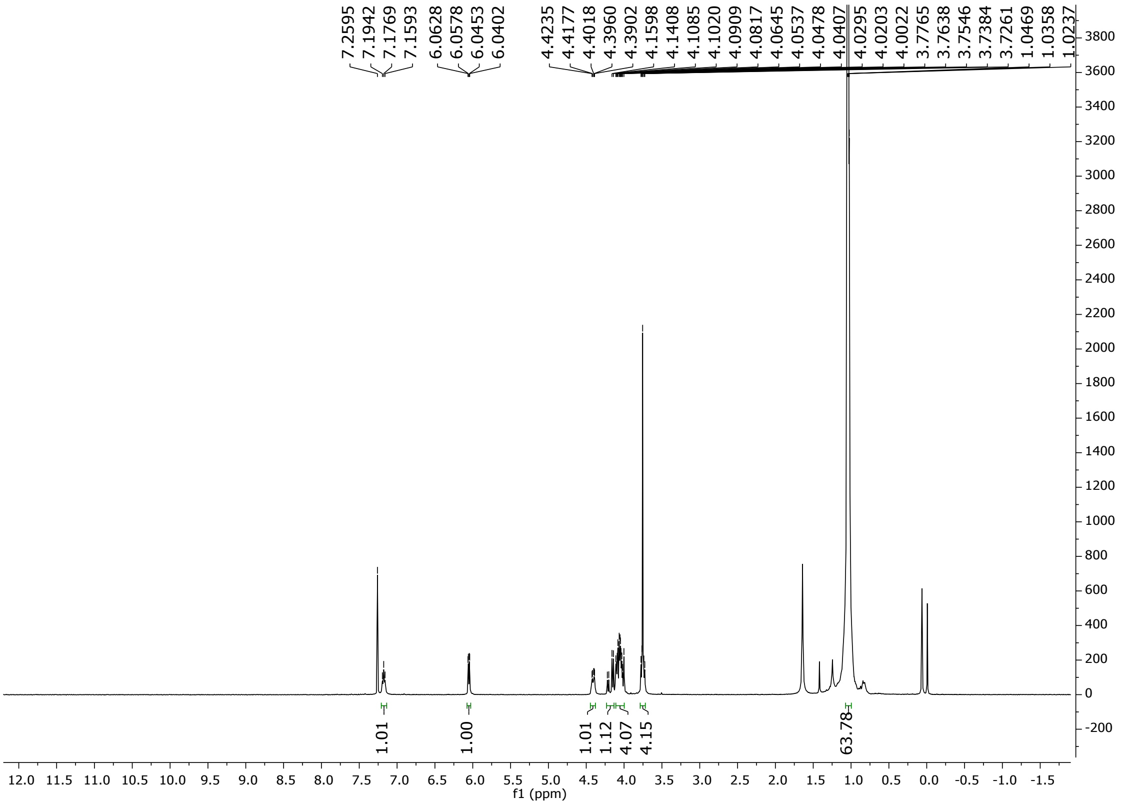

^{13}C NMR spectra (75 MHz, $CDCl_3$) of **3j**

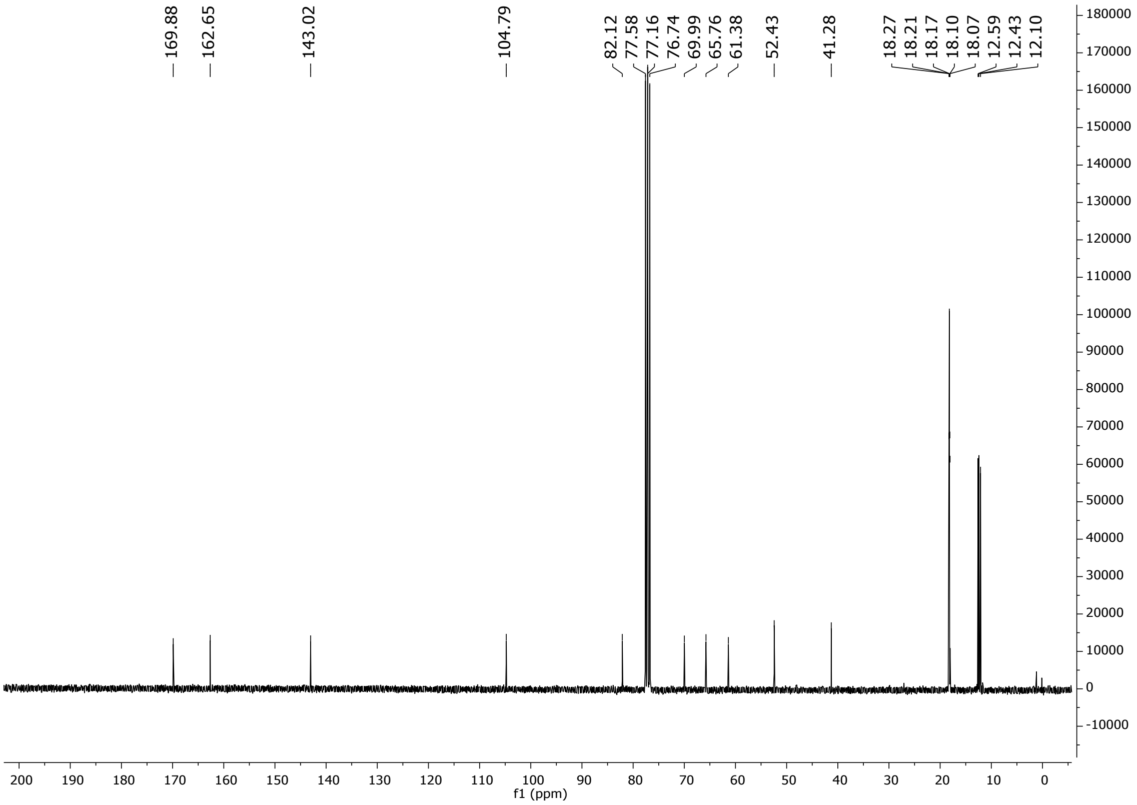

^{1}H NMR spectra (300 MHz, $CDCl_3$) of **3k**

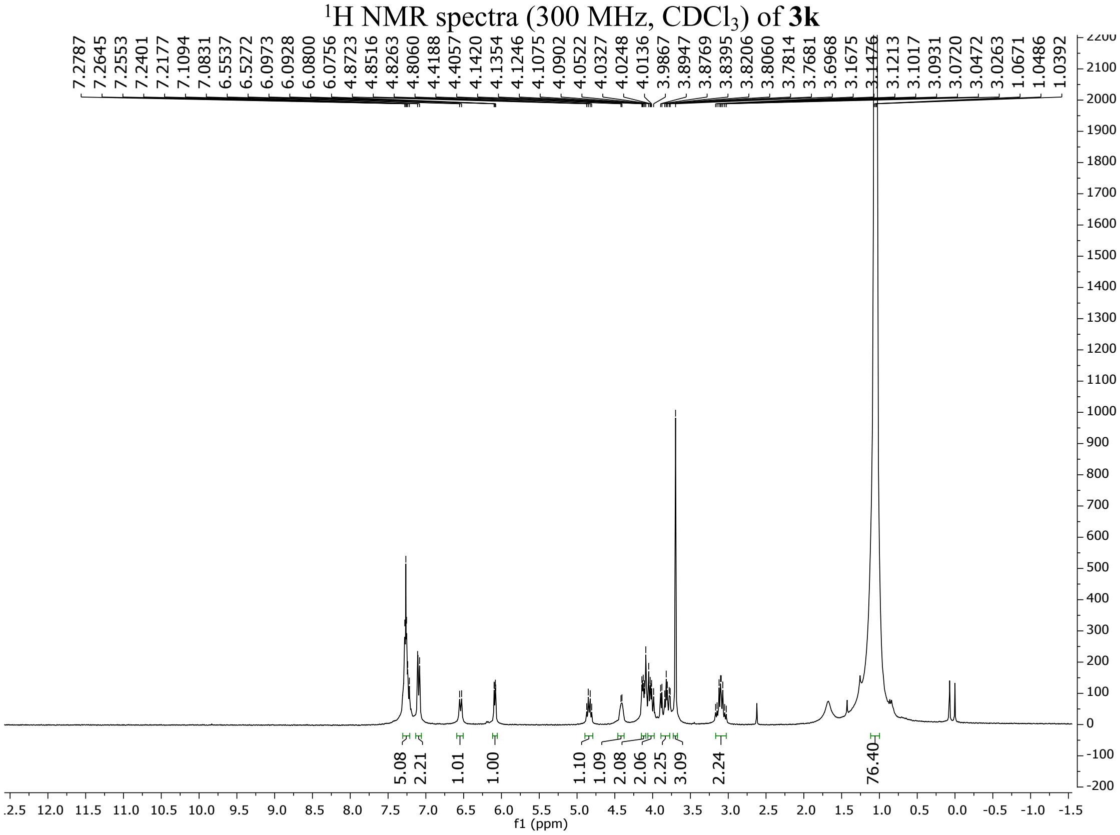

^{13}C NMR spectra (75 MHz, $CDCl_3$) of **3k**

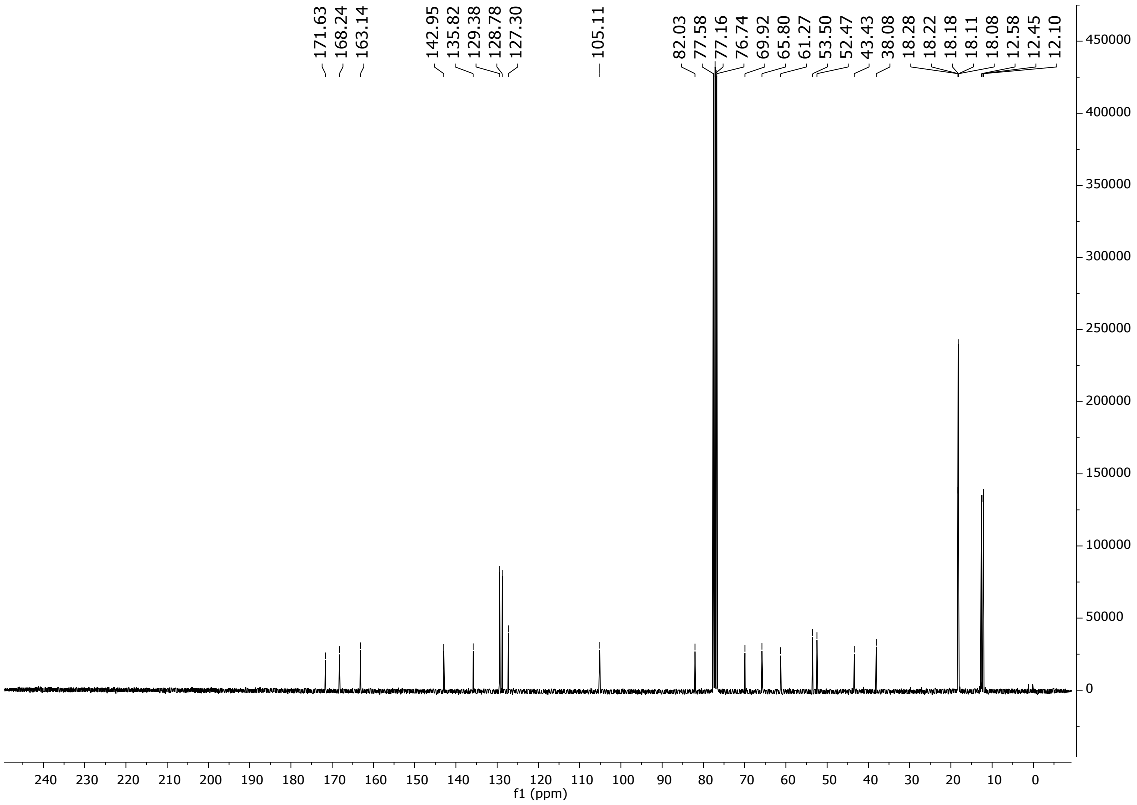

^1H NMR spectra (300 MHz, $CDCl_3$) of **3l**

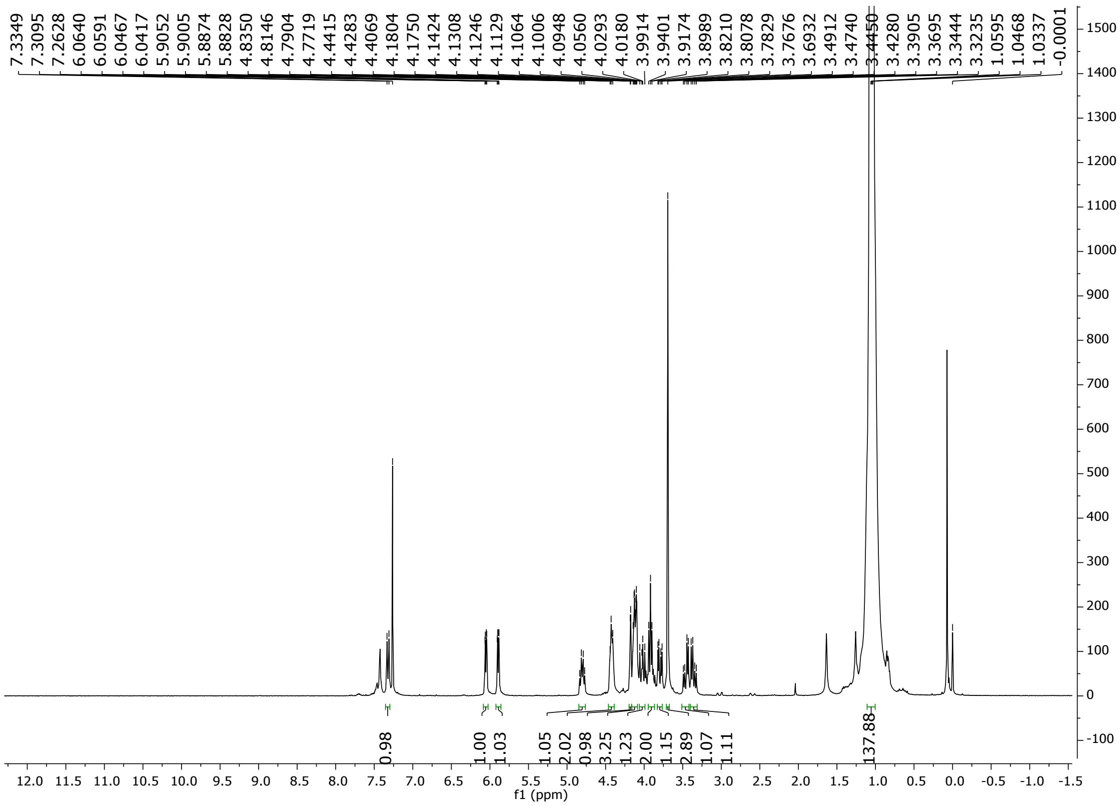

^{13}C NMR spectra (75 MHz, $CDCl_3$) of **3l**

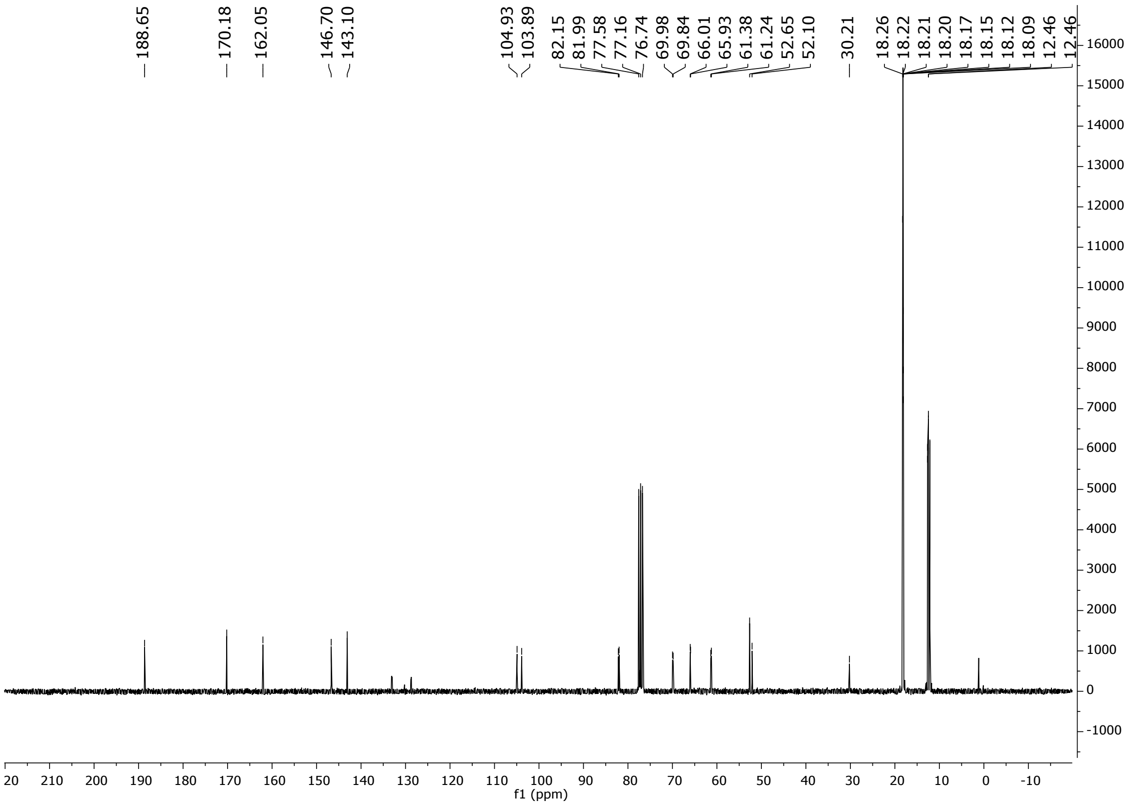

^{1}H NMR spectra (300 MHz, $CDCl_3$) of **3m**

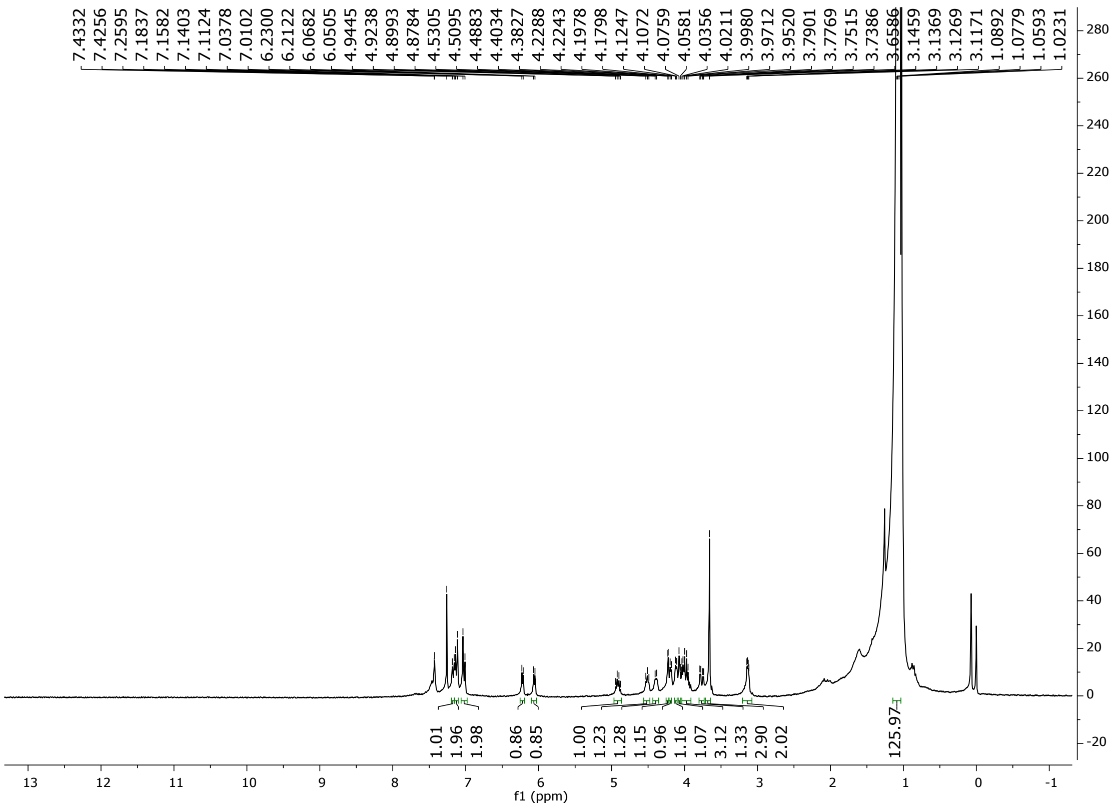

^{13}C NMR spectra (75 MHz, $CDCl_3$) of **3m**

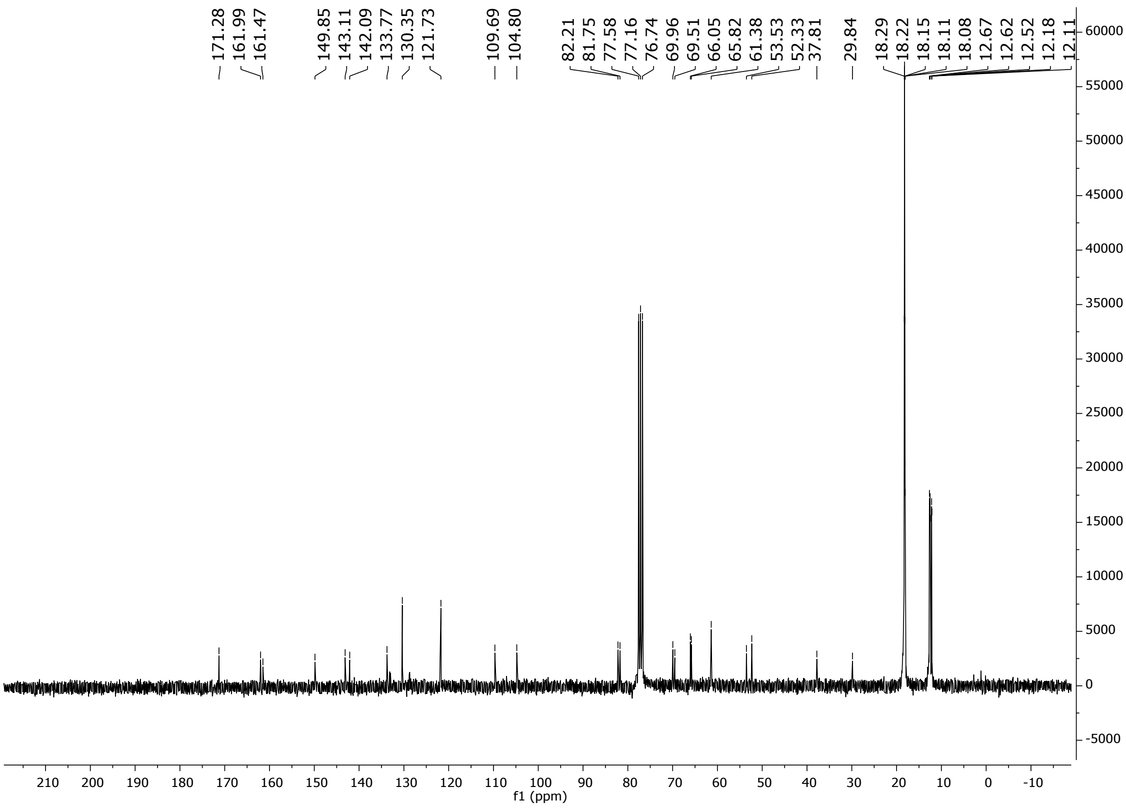

^{1}H NMR spectra (300 MHz, $CDCl_3$) of **3n**

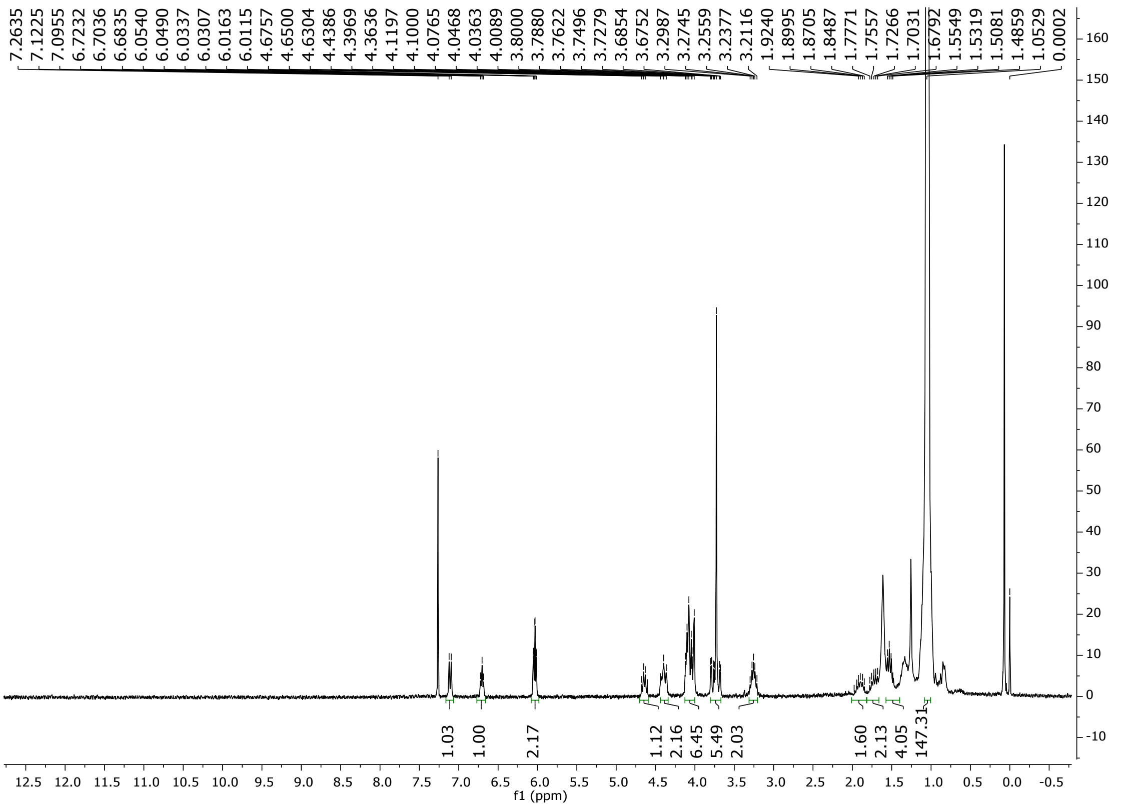

^{13}C NMR spectra (75 MHz, $CDCl_3$) of **3n**

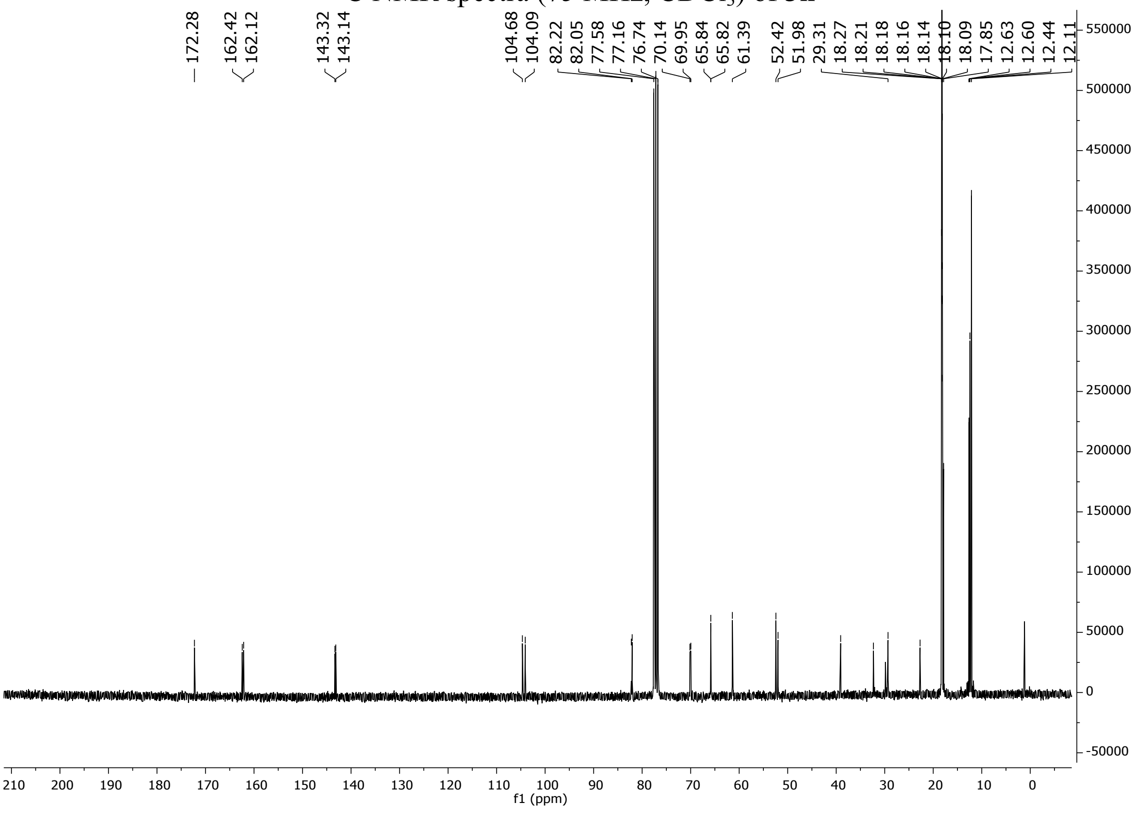

^{1}H NMR spectra (300 MHz, $CDCl_3$) of **3o**

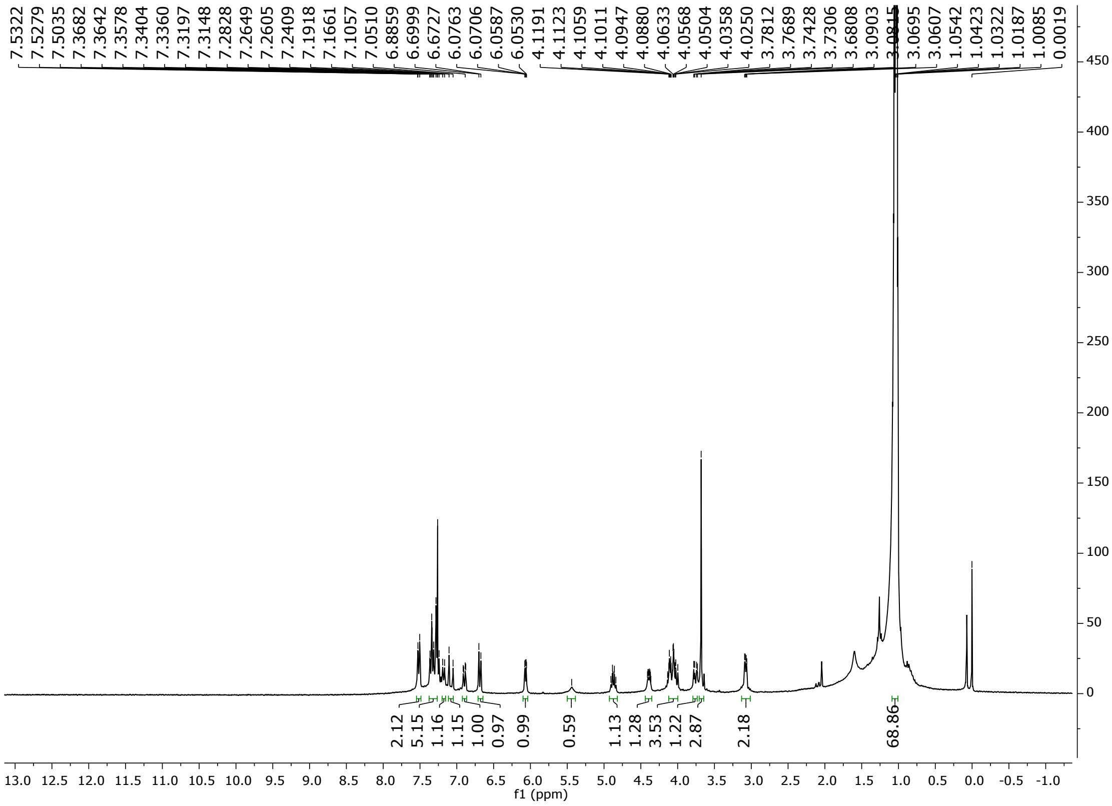

^{13}C NMR spectra (75 MHz, $CDCl_3$) of **3o**

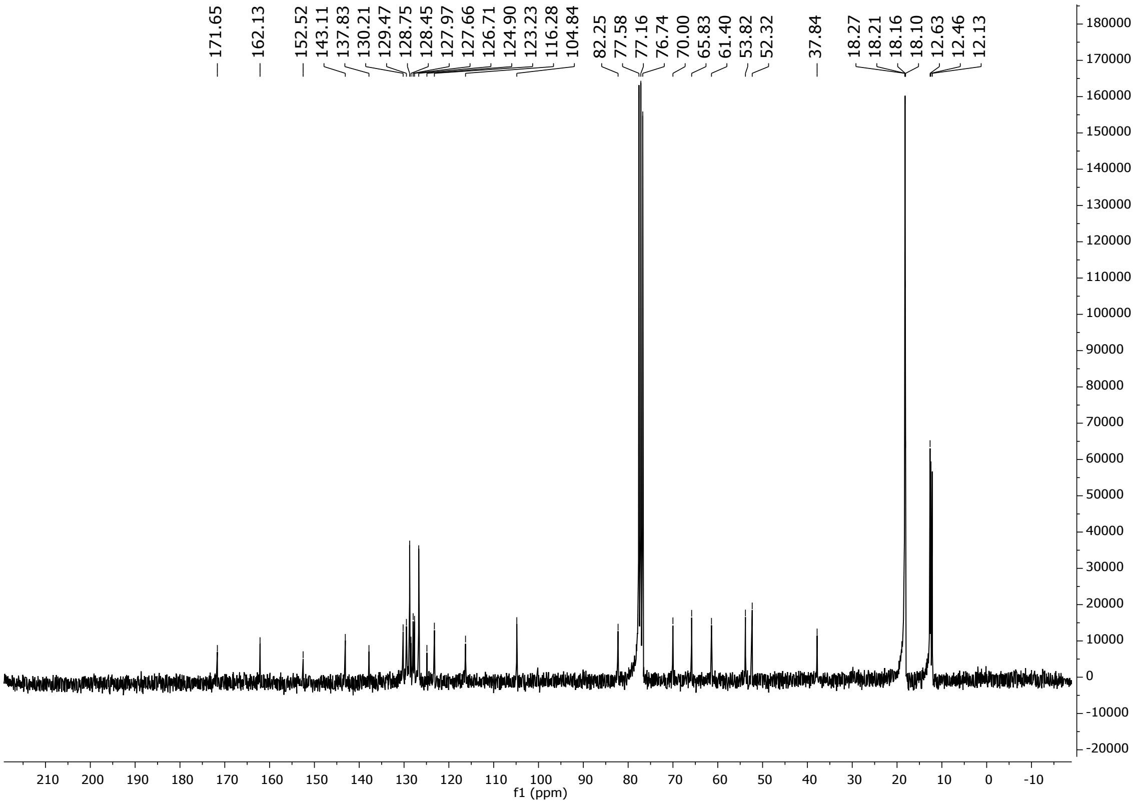

^{1}H NMR spectra (300 MHz, $CDCl_3$) of **3p**

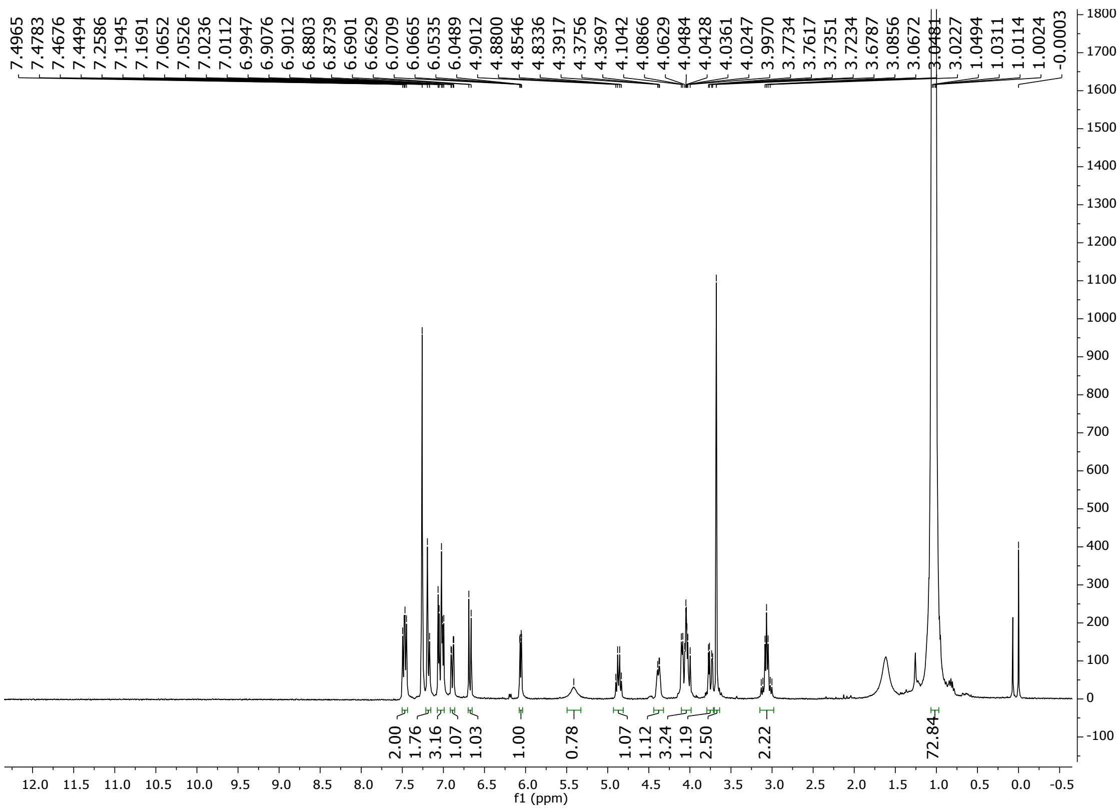

^{13}C NMR spectra (75 MHz, $CDCl_3$) of **3p**

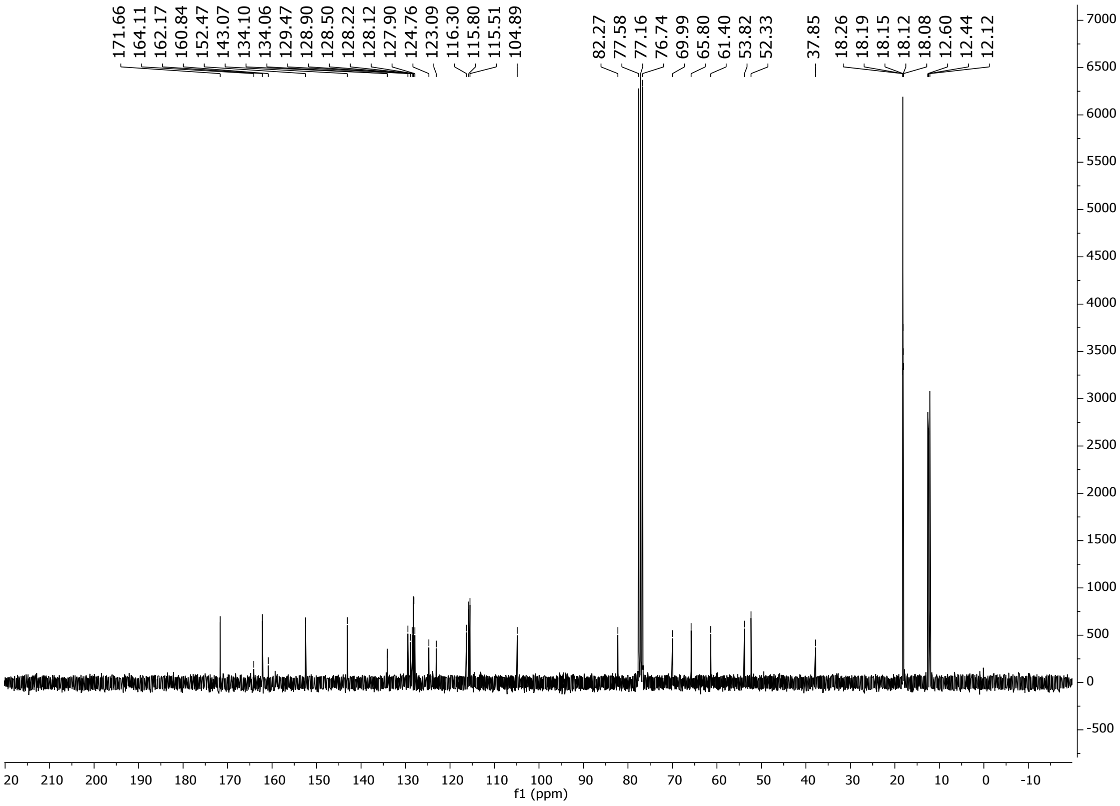

^{1}H NMR spectra (300 MHz, MeOD) of **4**

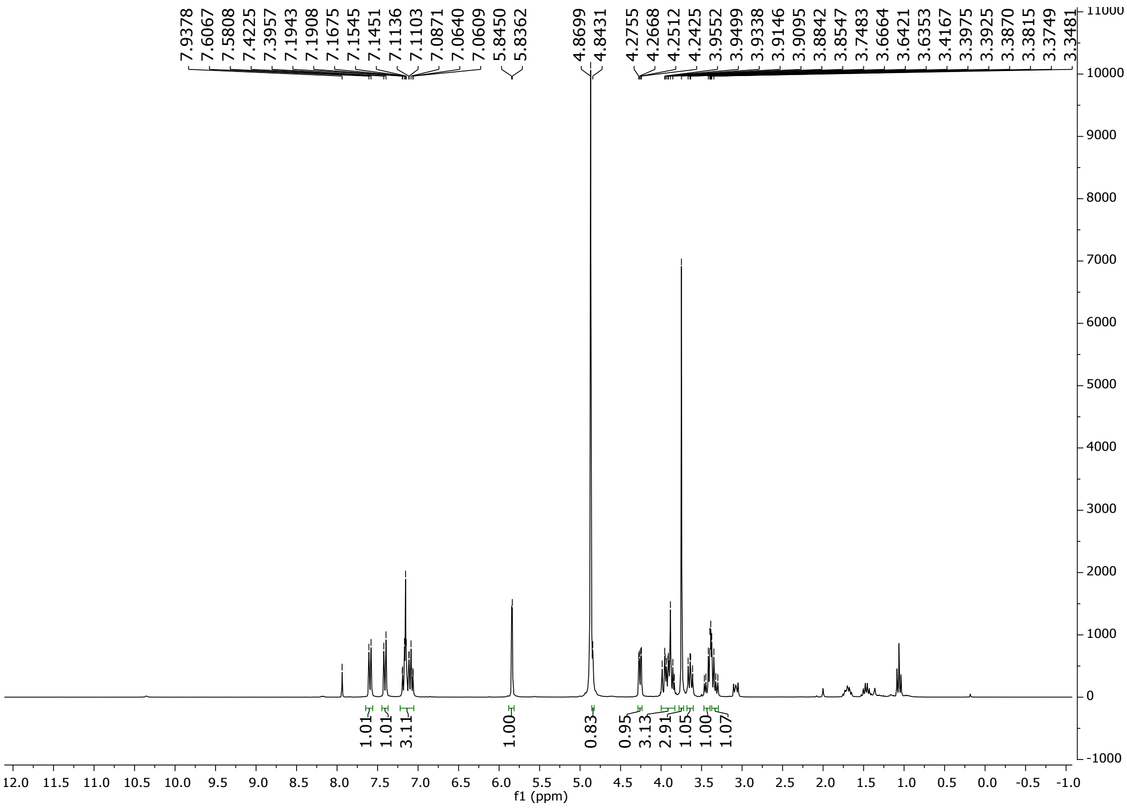

^{13}C NMR spectra (75 MHz, MeOD) of **4**

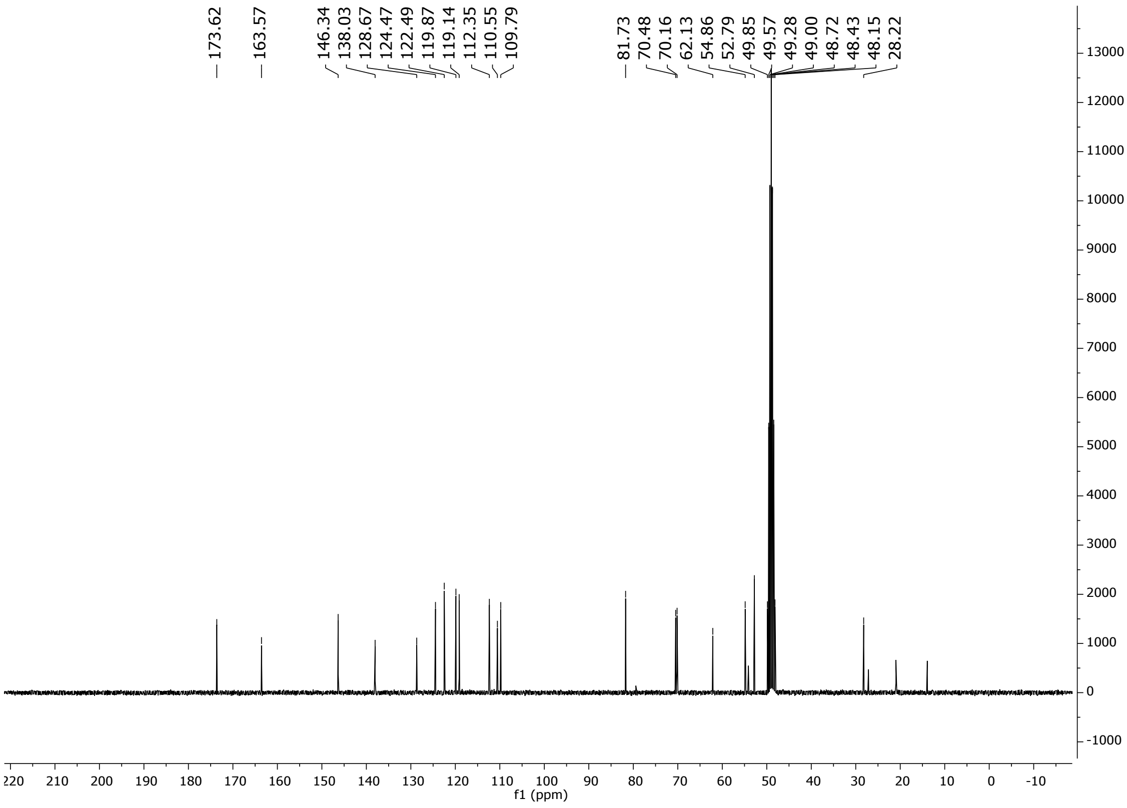

Supplement: Supplementary file 1 [file ao5c04865_si_001.pdf]
